# Mental health literacy training interventions for future health professionals: An evidence and gap mapping scoping review

**DOI:** 10.1371/journal.pone.0360403

**Published:** 2026-10-27

**Authors:** Rouwida ElKhalil, Noon Alrabee, Anuradha P. Gaydhanker, Wafaa El Zaatari, Imad Maalouf, Mohammed AlMaqbali, Yasir Ahmed Mohammed Elhadi, Moustafa Sherif, Aya Darwish, Messaouda Belfakir, Eny Qurniyawati, Abdulsalam El Mikkawi, Mohamad AlMekkawi

**Affiliations:** 1 Institute of Public Health, College of Medicine and Health Sciences, United Arab Emirates University, Al Ain, United Arab Emirates; 2 Zayed University, College of Interdisciplinary Studies, Dubai, United Arab Emirates; 3 Nursing Department, Fatima College of Health Sciences, Al Ain, United Arab Emirates; 4 Department of Anatomy and Embryology, Benha University, Banha, Egypt; 5 Department of Physiology, Benha University, Banha, Egypt; 6 Department of Epidemiology, Biostatistics, Population Studies, and Health Promotion, Faculty of Public Health, Universitas Airlangga, Surabaya, Indonesia; 7 Research group for health and wellbeing of women and children, Universitas Airlangga, Surabaya, Jawa Timur, Indonesia; 8 Faculty of Medicine, Tbilisi State Medical University, Tbilisi, Georgia; UoW: The University of Waikato, NEW ZEALAND

## Abstract

**Purpose:**

This study examined the global landscape of mental health literacy (MHL) education for health professions students, focusing on publication trends, intervention designs, pedagogical approaches, MHL domains addressed, evaluation methods, and contextual influences.

**Methods:**

Following PRISMA-ScR guidelines, an Evidence and Gap Mapping (EGM) scoping review was conducted to identify and visually synthesize MHL education interventions. Searches were conducted in PubMed, Embase, Scopus, Web of Science, EBSCOhost (including CINAHL, PsycINFO, and ERIC), and APA PsycArticles (February 1997-May 2025). The included studies involved pre-licensure health professions students and implemented structured educational interventions explicitly targeting MHL domains. The data were extracted using a standardized framework and analyzed through descriptive synthesis and visual mapping to identify research clusters and gaps. PROSPERO registration: CRD420251050891.

**Results:**

Fifty-five studies met the inclusion criteria for this review. Notably, the number of publications increased significantly after 2016, with most of the evidence coming from Europe, the Western Pacific, and the Americas, while representation from the African and Eastern Mediterranean regions was limited. Quasi-experimental designs were the most common, characterized by short-term, classroom-based interventions primarily targeting specific professions, particularly nursing and medicine. Although traditional didactic teaching methods remained prevalent, experiential strategies such as simulation, role-play, and reflective practices were increasingly adopted. Within the existing body of evidence, interventions predominantly emphasized knowledge, attitudes, and confidence, whereas skills development, behavioral change, self-awareness, and help-seeking were less consistently addressed. Furthermore, evaluations mostly relied on short-term, self-reported outcomes, with few studies examining behavioral change or long-term impacts.

**Conclusions:**

MHL education for future health professionals is expanding but remains unevenly distributed, pedagogically conservative, and methodologically limited. Advancing the field will require longitudinal, theory-informed, and experiential approaches, stronger evaluation designs, integration of lived-experience perspectives, and targeted capacity-building in low- and middle-income settings.

## Introduction

The rising prevalence of mental disorders across socioeconomic and demographic groups has become a major global public health concern [1]. The World Health Organization (WHO) defines mental health as a state of mental well-being that allows people to handle life’s stresses, realize their abilities, learn, work effectively, and contribute to their community [2]. Worldwide estimates suggest that one in four people will experience a mental or neurological disorder at some point in their lives, underscoring the widespread impact of mental health challenges [3]. This burden is particularly pronounced among young people: one in seven adolescents aged 10–19 is affected by a mental disorder, with depression, anxiety, and behavioral disorders among the leading contributors to morbidity in this age group [4].

A growing body of research has examined the factors underlying the high incidence of mental disorders [1,3,5,6]. One consistently identified challenge is the low level of mental health literacy (MHL) in the general population, which has been associated with difficulties in recognizing mental health problems, increased stigma, and delayed help-seeking and treatment utilization [5,7–10]. MHL encompasses the knowledge, attitudes, and beliefs that support the recognition, management, and prevention of mental disorders [8]. Within healthcare, improving MHL is especially important as healthcare providers frequently encounter patients experiencing mental health concerns. Yet, numerous studies highlight substantial gaps in training, confidence, and competency in this area [10]. These gaps contribute to stigma, misdiagnosis, and inadequate treatment [11]. Despite growing recognition of the importance of MHL within health professions education, substantial variability persists in how such training is designed, delivered, and evaluated [12]. Previous reviews have contributed important evidence on MHL interventions across school, community, digital, and healthcare settings. For example, reviews of school- and youth-focused interventions have examined effects on knowledge, stigma, and help-seeking outcomes [13], while more recent reviews have explored web- and app-based approaches to improving MHL [14,15]. In health-related contexts, systematic and realist reviews have examined mental health education and MHL interventions among healthcare professionals and health professions students, highlighting the importance of experiential learning, stigma reduction, and context-sensitive educational design [12,16]. However, these reviews have generally focused on intervention effectiveness, specific populations, or particular delivery modalities rather than systematically mapping the global distribution, pedagogical characteristics, theoretical foundations, implementation factors, and evaluation approaches of MHL education for pre-licensure health professions students. Improving MHL during undergraduate training may enhance future clinicians’ ability to recognize and respond to mental health problems in patients. It may also promote positive attitudes toward mental health, reduce stigma, and encourage appropriate help-seeking among students themselves [17].

This study addresses this gap by conducting an Evidence and Gap Mapping (EGM) study to systematically map, categorize, and visually synthesize the global evidence on MHL education for health professions students. The aim was to characterize the landscape of published studies, identify clusters of evidence and gaps, and highlight areas requiring further research. Although previous systematic, scoping, and realist reviews have examined the effectiveness of MHL interventions or specific educational approaches across school, community, digital, and healthcare settings [12–14,16], they have primarily focused on intervention effectiveness, particular populations, or specific delivery modalities. Consequently, limited attention has been given to how MHL education is distributed across health professions, geographical regions, pedagogical approaches, theoretical frameworks, implementation contexts, and evaluation strategies. The present review addresses this gap by providing, to our knowledge, the first evidence and gap map specifically focused on MHL education for pre-licensure health professions students. By integrating educational design characteristics, implementation factors, and outcome evaluation within a single analytical framework, this review provides a comprehensive overview of the current evidence base and identifies priorities for future research, curriculum development, and policy. The specific objectives were to:

1. Map the scope and distribution of the literature, including publication trends, geographical coverage, and methodological characteristics.
2. Categorize and map the design and context of MHL interventions, including target student populations, core content, pedagogical strategies, and underlying theoretical frameworks.
3. Map and identify gaps in assessment approaches and implementation factors by aligning measured outcomes with a training evaluation framework and synthesizing reported barriers and facilitators.

## Methods

### Design

This study utilized an evidence and gap mapping (EGM) scoping review approach to systematically catalogue and visualize the evidence base on MHL education for health professions students. Unlike a traditional systematic review, which primarily focuses on synthesizing evidence regarding intervention effectiveness, an EGM study aims to map the distribution of evidence across a field and identify research gaps [18,19].The EGM approach involved categorizing MHL educational interventions across key analytical dimensions, including health professions, geographical regions, intervention characteristics, targeted MHL domains, and evaluation outcomes. Evidence was synthesized using cross-tabulation matrices and graphical displays to illustrate areas of evidence concentration and scarcity. The study was guided by established methodological frameworks for evidence and gap mapping [18,19], and its conduct and reporting adhere to the Preferred Reporting Items for Systematic reviews and Meta-Analyses extension for Scoping Reviews (PRISMA-ScR) [20]. The review protocol was registered with the International Prospective Register of Systematic Reviews PROSPERO (CRD420251050891).

### Eligibility criteria

The inclusion criteria were established according to the PCC (Population, Concept, Context) model, which is recommended for scoping and evidence- and gap-mapping studies. The population of interest comprised students enrolled in undergraduate or postgraduate programs in the health professions, including medicine, nursing, pharmacy, dentistry, psychology, midwifery, and allied health. For the purposes of this review, health professions students were defined as individuals enrolled in pre-licensure education programs preparing for professional practice in these disciplines. Postgraduate students were defined as learners enrolled in formal academic postgraduate programs; residents, interns, and other postgraduate trainees in clinical service-based programs were excluded. Given international variation in educational structures, undergraduate and postgraduate classifications were based on the terminology used in the original studies. Studies involving licensed practitioners engaged in continuing education or students from non-health disciplines were excluded. The central concept under investigation was any structured educational intervention with an explicit objective to enhance MHL, informed by established conceptual frameworks [8,10]. Interventions were required to target one or more core MHL domains: knowledge (e.g., of disorders and treatments), attitudes (e.g., stigma reduction), skills (e.g., communication, screening), or behaviours (e.g., help-seeking, referral). Studies were eligible if they described a distinct, structured educational intervention or a clearly delineated curriculum module with an explicit objective to improve one or more MHL domains. General curricula were included only when a mental health-specific component was separately identifiable in terms of content, delivery, and outcomes. The context was deliberately broad, with no restrictions on geographical location, educational setting, or the presence of a comparator group. To be included, studies had to report at least one relevant outcome or process measure to verify the intervention’s implementation. However, the type or significance of the outcome was not a criterion for filtering. All primary research designs, including quantitative, qualitative, and mixed methods approaches, were eligible for consideration. Secondary literature, non-peer-reviewed material, opinion pieces, theses, and conference abstracts were systematically excluded from the analysis. Only studies that had undergone peer review and quality control were included. The search window spanned from 17 February 1997, the date of the first paper introducing the concept of mental health literacy [9], to 1 May 2025. Searches were limited to English-language publications.

### Information sources and search

A comprehensive search was conducted in PubMed, Embase, Scopus, Web of Science, EBSCOhost (including CINAHL, PsycINFO, and ERIC), and APA PsycArticles (via ProQuest). The search strategy was developed by an academic librarian and combined controlled vocabulary (e.g., MeSH) and keywords for three concepts: (1) health professions students, (2) MHL, and (3) education/training (S 1A in S1 File). Reference lists of included articles were hand-searched. Although PsycINFO (via EBSCOhost) and APA PsycArticles (via ProQuest) contain some overlapping content, both databases were searched to maximize retrieval of relevant studies. Any duplicate records identified across databases were removed during the deduplication process in Covidence before screening.

### Study selection

Search results were imported into Covidence for deduplication and screening. In the first stage, two reviewers (AD and MB) independently screened titles and abstracts against the predefined PCC criteria. In the second stage, full-text articles were independently assessed by NA and WE. Any discrepancies were resolved through consensus or, when necessary, settlement by a third reviewer (MS for title and abstract screening and RE for full-text review). The selection process was documented using a PRISMA flow diagram

### Data charting

A standardized data charting form was developed in Microsoft Excel for Microsoft 365 and subsequently tested for clarity and consistency across five randomly selected studies. Two reviewers independently extracted data from all included studies using this standardized form. Any discrepancies were addressed through structured consensus discussions, with a third reviewer involved when necessary to reach an agreement. Inter-coder consistency was assessed iteratively during pilot testing and after each extraction, allowing refinements to the coding framework to ensure consistent interpretation of variables and minimize bias. The final form captured comprehensive study-level information across six domains: (1) Study characteristics (author(s), year, journal, country, WHO region [21], study context, design, and setting); (2) Participant characteristics (health profession(s), education level, sample size, and sampling method); (3) Intervention characteristics (name, type, duration, delivery mode, and facilitator category, including instructional strategies and technology use); (4) MHL components (targeted domains and key mental health topics); (5) Evaluation metrics (assessment method, timing, assessor category, instruments used, Kirkpatrick level [22], and key findings; and (6) Qualitative findings about barriers and facilitators. The original Kirkpatrick framework categorizes educational outcomes into four levels: reaction, learning, behavior, and results [22]. For the purposes of evidence mapping, Level 2 (Learning) outcomes were further subdivided by the review team into attitude/stigma (2a), knowledge (2b), and confidence/self-efficacy (2c) to enable more granular classification of the outcomes reported across studies. These subcategories represent an analytical adaptation and are not formal components of the original Kirkpatrick framework. This structured form enabled consistent data entry, facilitating coding, cross-tabulation, and pattern visualization across studies, professions, regions, and outcomes. Studies addressing multiple MHL topics were coded multiple times but counted only once in the overall tallies.

### Classification and coding framework

The extracted data were organized using a structured classification framework based on established evidence-mapping methodologies [18,19]. This framework enabled systematic coding, categorization, and comparison of study characteristics across multiple analytic dimensions. The coding process primarily followed a deductive approach, using predefined variables from the literature, complemented by inductive refinements to capture emerging patterns during data extraction.

Each study was coded across several standardized dimensions, including study design, sampling method, participant and intervention characteristics, MHL domains addressed, contextual factors, and evaluation metrics. Standardized code definitions ensured consistency across reviewers, drawing from existing health education research frameworks. The finalized coding schema included variables like intervention type, duration, delivery mode, facilitator category, educational setting, outcome domain, and assessor type. Qualitative data on implementation factors, such as barriers and facilitators, were also coded for emergent themes. To ensure reliability and transparency, all codes were compiled in a unified coding template, with definitions documented in a standardized codebook (S 1B in S1 File). This approach enabled thorough mapping of evidence and facilitated cross-tabulation analyses to identify relationships and gaps across the defined categories.

In alignment with the evidence and gap mapping methodology, coded variables were systematically organized into structured matrices that cross-tabulated key dimensions of interest. These dimensions included, for example, MHL domains by health profession, intervention characteristics by geographical region, and outcome categories corresponding to Kirkpatrick evaluation levels. The cells within these matrices indicated the number of studies contributing evidence for each specific intersection. Areas with few or no studies, especially in underrepresented regions, at advanced evaluation levels, or in less frequently targeted MHL domains, were identified as evidence gaps. To promote transparency and reproducibility, all coding decisions were guided by predefined operational definitions outlined in a shared codebook. Any ambiguities encountered during the extraction process were resolved through team discussions, with documented revisions made to the coding schema.

### Data synthesis and visualization

The data synthesis was primarily descriptive, focusing on the characteristics and distribution of evidence on MHL education for health profession students. Consistent with the EGM approach, the synthesis prioritized visual mapping of evidence distribution rather than effect estimation. This approach facilitated the identification of research clusters and gaps across various intervention types, contexts, and outcome domains. In accordance with the EGM methodology, no meta-analysis or quality appraisal was performed. Data were summarized using frequency counts and percentages to illustrate trends in publication patterns, study designs, participant characteristics, intervention components, and evaluation strategies.

Findings were presented narratively and visually, using line graphs for publication trends and bar charts for study designs, settings, intervention durations, and participant distributions by profession and educational level. Tables detailed instructional strategies, technology integration, experiential components, theoretical frameworks, and assessment instruments. Population data were analyzed by discipline to reflect the multi-professional nature of the interventions. Summary matrices mapped outcomes to Kirkpatrick evaluation levels, accounting for evaluation timing and follow-up. This synthesis provided an overview of research activity and gaps in MHL education for future health professionals. Additionally, contextual factors affecting implementation were analyzed thematically, yielding insights into the environmental, institutional, and logistical influences on MHL educational outcomes. This integration ensured quantitative trends were understood alongside the real-world conditions that affect program feasibility and scalability.

All descriptive analyses and figures were generated using Microsoft Excel for Microsoft 365 (Microsoft Corp., Redmond, WA, USA). Frequency counts and percentages were calculated directly from the standardized data extraction spreadsheet. Figures were created using Excel’s charting functions, with Microsoft Copilot used to assist in chart formatting and layout refinement for publication. The underlying datasets used to generate all figures are provided in the S2 File.

### Unit of analysis and management of multiplicity

This analytical structure was complemented by clear rules for managing study-level multiplicity and ensuring consistent counting across categories. The primary unit of analysis was the individual study, consistent with guidance on scoping and evidence-mapping reviews [23,24]. Multiple publications reporting on the same underlying study population were collated and treated as a single study to prevent double-counting.

For analyses of educational settings, intervention characteristics, instructional components, mental health literacy domains, outcomes, and contextual factors, studies were permitted to be coded into more than one category where applicable. Accordingly, a single study that implemented multiple components or addressed multiple domains was counted once for each relevant category. Category totals and percentages, therefore, reflect non–mutually exclusive coding and may exceed the total number of included studies. Percentages were calculated either using the total number of included studies (n = 55) or the total number of coded occurrences, as specified in the corresponding figure caption or table note. However, each study was counted only once in the overall study-level tallies.

## Results

A comprehensive search across six electronic databases identified 3,461 records, with an additional four records retrieved through reference-list searching, for a total of 3,465 records. After removing 300 duplicate records using Covidence, 3,165 titles and abstracts were screened, of which 3,040 were excluded. The remaining 125 full-text articles were assessed for eligibility. Seventy studies were subsequently excluded because of an ineligible population (n = 8), intervention (n = 58), or study design (n = 4). Consequently, 55 studies met the inclusion criteria and were included in the evidence and gap map (Fig 1) (S 1C in S1 File).

**Fig 1:**
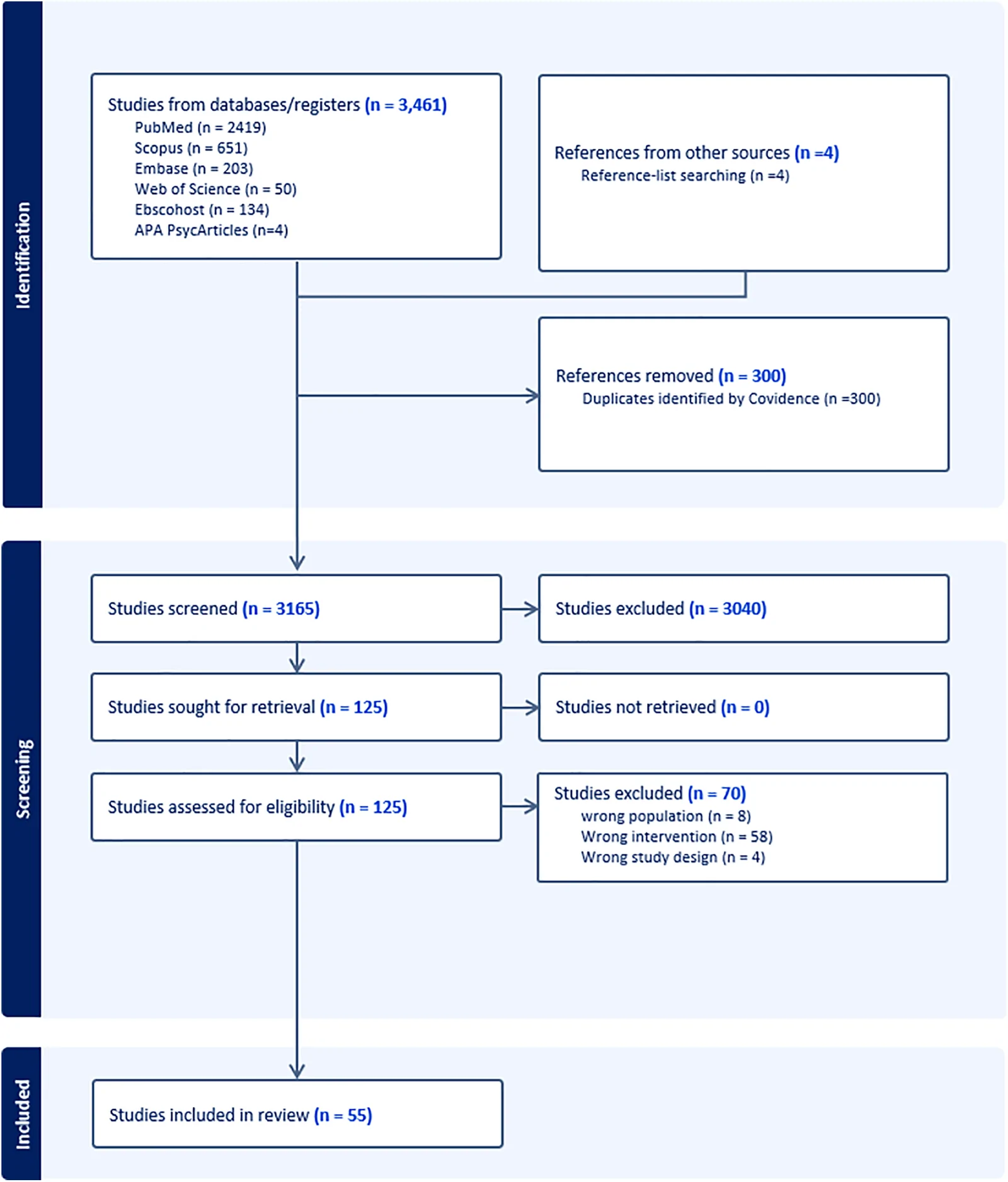
The PRISMA flow diagram.

### Timeline characteristics

A total of 55 studies were published between 1997 and 2025 (Fig 2). The earliest included study was published in 1999, with relatively few publications during the early 2000s. Publication output increased steadily after 2016 and peaked in 2022, when eight studies were published, representing the highest annual output during the review period. Although publication numbers declined slightly thereafter, research activity remained relatively high, with seven studies published in 2023 and additional studies identified through 2025.

**Fig 2:**
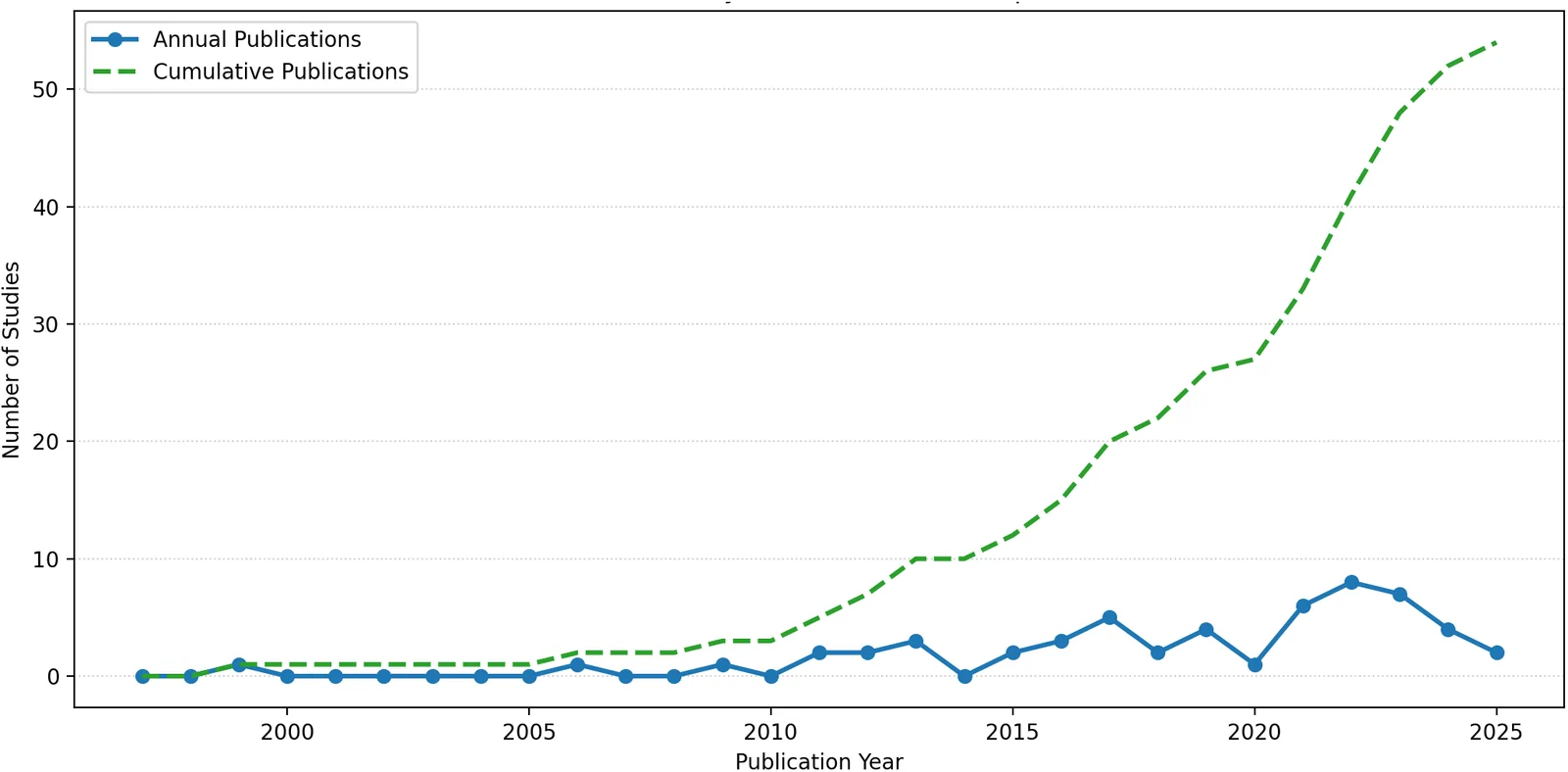
Publication trends in mental health literacy education for health professions students (1997–2025). The Fig. illustrates a sharp increase in publication activity after 2016.

### Regional characteristics

The included studies showed substantial regional variation in publication output (Fig 3A). The European Region (EUR) and Western Pacific Region (WPR) accounted for the largest shares of publications (n = 19 and n = 16), with the United Kingdom, Australia, and Malaysia contributing the most studies within these regions. The Region of the Americas (AMR) contributed 14 studies, with notable contributions from the United States and Brazil. In contrast, the Eastern Mediterranean (EMR) and African Region (AFR) were severely underrepresented, with only 4 and 2 studies, respectively. No eligible studies were identified from the WHO South-East Asia Region (SEAR), resulting in an uneven geographical distribution of the available evidence. Studies from EUR and WPR predominantly assessed knowledge- and stigma-related outcomes and employed quasi-experimental or randomized designs, whereas evidence from EMR and AFR focused mainly on foundational outcomes, particularly knowledge and attitudes/stigma. Only one EMR study addressed mental health and wellbeing, and no studies from either region evaluated skills or self-efficacy (S 1D in S1 File). Overall, the mapped evidence was concentrated in Europe, the Western Pacific, and the Americas, whereas substantially fewer studies originated from the Eastern Mediterranean and African regions (S 1D in S1 File).

**Fig 3:**
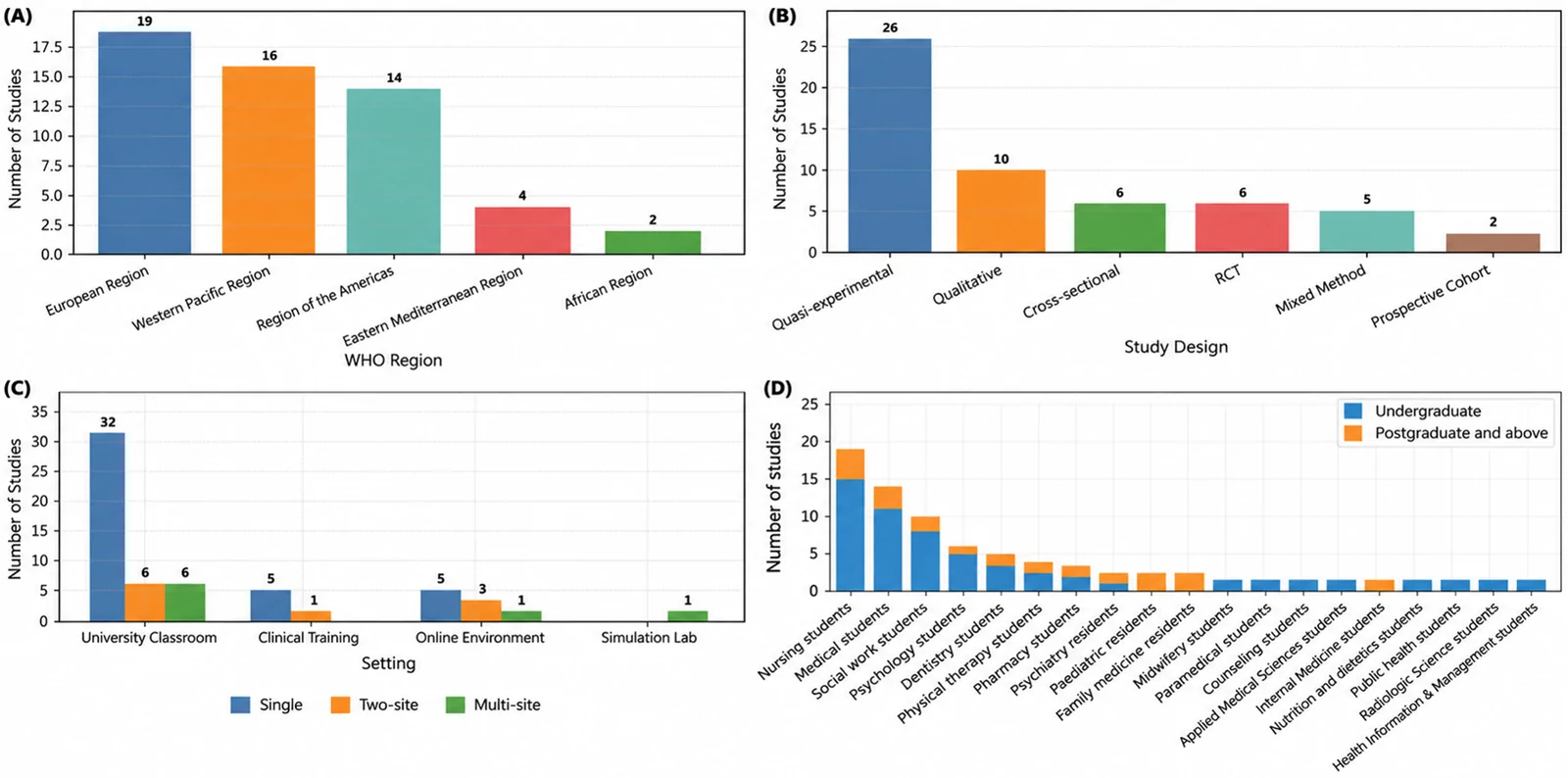
Study characteristics in mental health literacy education for health professions students. (A) Number of included studies by World Health Organization (WHO) region (n = 55 studies). (B) Distribution of study designs across included studies (n = 55 studies). (C) Distribution of study settings by site context (single-site, two-site, and multi-site) (non-mutually exclusive setting counts). (D) Distribution of included studies by health profession and educational level (undergraduate vs postgraduate and above) (n = 55 studies). Panels C and D include non-mutually exclusive categories; studies could contribute to more than one applicable category.

### Study characteristics and context

Beyond the geographical distribution of studies, important patterns also emerged in their methodological characteristics and educational contexts. The analysis grouped study designs into six categories (Fig 3B): quasi-experimental (n = 26), qualitative (n = 10), RCTs (n = 6), mixed methods (n = 5), cross-sectional (n = 6), and cohort (n = 2). Quasi-experimental designs were the most frequently used study design, accounting for nearly half of the included studies. Among quasi-experimental studies, 21 of 26 (80.8%) included a control group, while all RCTs (6/6, 100%) incorporated control conditions by design; control groups were less common in observational studies (3/8, 37.5%).

Study settings were predominantly university-based (Fig 3C). Among single-site settings, university classrooms were most common (n = 32), followed by clinical training settings (n = 5) and online environments (n = 5). Two-site studies were conducted in university classroom settings (n = 6) and online environments (n = 3). Multi-site studies included university classrooms (n = 6), clinical training settings (n = 1), online environments (n = 1), and simulation laboratories (n = 1). Because some studies involved more than one educational setting, setting categories were not mutually exclusive, and the total number of setting counts exceeded the number of included studies. Overall, university classroom settings predominated across site configurations, whereas simulation laboratory settings were rarely represented.

Sample sizes varied widely across study designs. Quasi-experimental studies typically enrolled 20–300 participants, with a small number exceeding 400 students. RCTs generally involved 55–300 participants, mixed-methods studies ranged from 24 to just over 200 participants, and observational studies showed the greatest variability (60 to more than 300 participants), highlighting substantial heterogeneity in sampling (S 1E in S1 File). Overall, the mapped evidence was characterized by predominantly institution-based, quasi-experimental studies, with relatively few multi-site collaborations or randomized study designs.

### Population characteristics

The methodological profile of the included studies was accompanied by clear patterns in the health professions and learner groups represented. Most interventions targeted undergraduate students from a single health profession and placed a strong emphasis on uniprofessional training models. Nursing students were the most frequently represented participant group (n = 19, 34.5%), followed by medical students (n = 14, 25.5%). Other health professions, including pharmacy, psychology, social work, dentistry, midwifery, and various allied health fields, were represented to a lesser extent, often appearing within smaller subsets of studies or as part of interprofessional cohorts. Fig 3D reflects the number of studies in which each professional group was represented, rather than identifying a single primary population per study. When studies included more than one profession, we counted each relevant professional group.

Approximately one quarter of studies employed interprofessional formats, which typically brought together students from two or more professions. Overall, the mapped evidence was concentrated in undergraduate nursing and medical education, with comparatively fewer studies involving other health professions or interprofessional educational models.

### Intervention characteristics

The evidence base also demonstrated considerable variation in how MHL interventions were designed, delivered, and facilitated across educational settings. The analysis of intervention durations showed that most interventions were very short-term (n = 28, 50.9%), followed by extended/continuous interventions (n = 10, 18.2%) and medium-term interventions (n = 9, 16.4%). Short-term interventions accounted for 4 studies (7.3%), while long-term interventions accounted for 2 studies (3.6%). Duration was not reported in 2 studies (3.6%) (Fig 4A). Overall, intervention duration was predominantly very short-term, with relatively few studies reporting extended or continuous delivery. The cumulative number of studies incorporating experiential components and educational technology increased after 2016 (S 1F in S1 File). Instructional strategies relied heavily on traditional methods, with didactic teaching/lectures (n = 32, 19.3%) being the most frequently reported, followed by group discussions/peer learning (n = 28, 16.9%) and case-based/problem-based learning (n = 25, 15.1%) (Fig 4B). Role-play/simulation-based learning accounted for 20 occurrences (12.0%), while video/multimedia learning accounted for 11 (6.6%) and e-learning/online platforms for 9 (5.4%). Patient/service-user involvement represented 5 occurrences (3.0%), whereas human rights/legal/ethics content accounted for 3 (1.8%). Percentages were calculated using the total number of coded instructional-strategy occurrences (N = 166) as the denominator. Because individual studies could employ more than one instructional strategy, these categories were not mutually exclusive.

**Fig 4:**
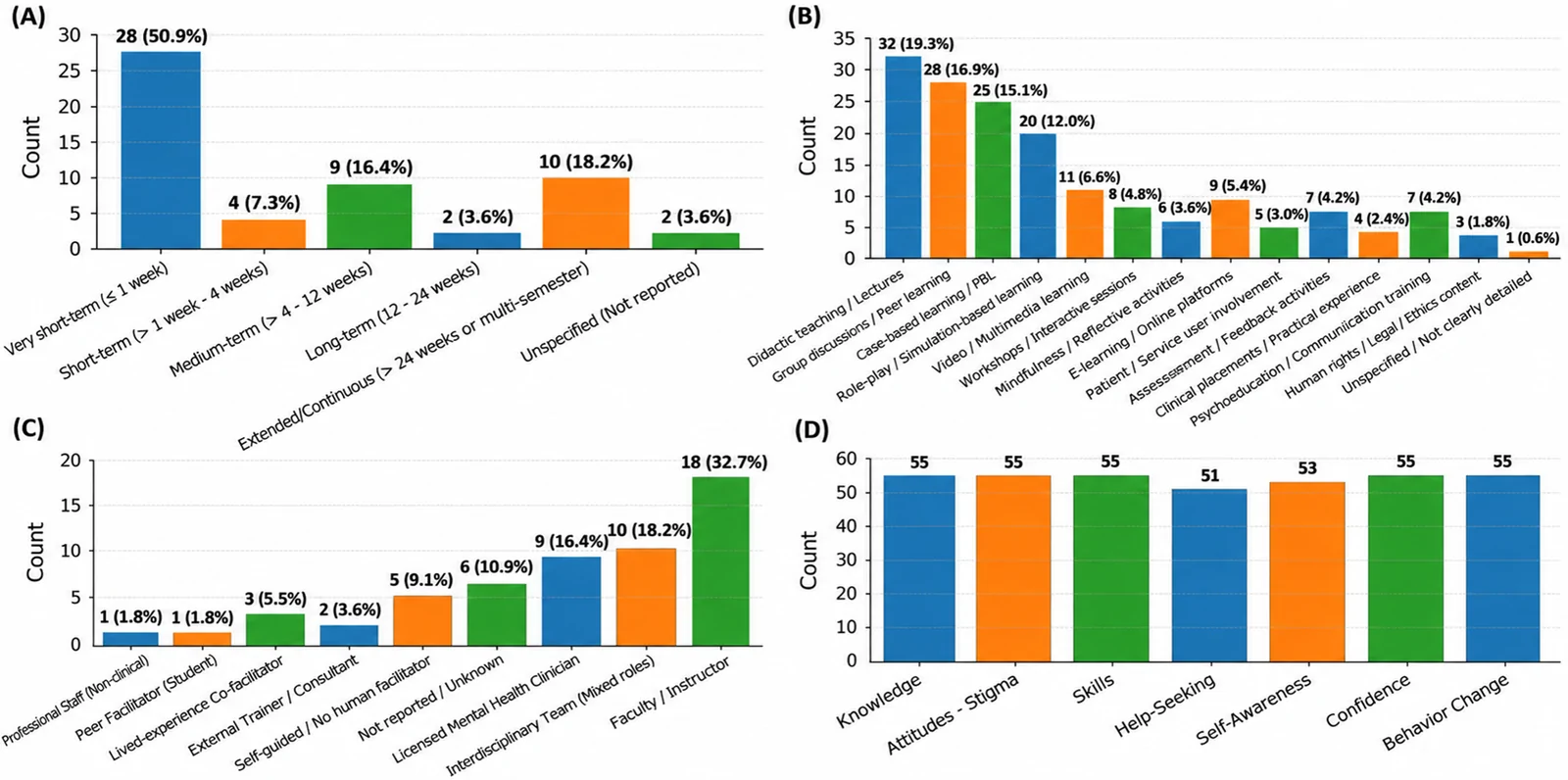
Characteristics of mental health literacy (MHL) educational interventions for health professions students. (A) Distribution of intervention duration categories (n = 55 studies). (B) Frequency of instructional component categories used across interventions (n = 166 coded occurrences). (C) Distribution of intervention facilitators by facilitator type (n = 55 studies). (D) Coverage of mental health literacy domains across included studies (n = 55 studies). Panels B and D include non-mutually exclusive categories and therefore may sum to more than 100%.

Facilitation was most often delivered by faculty or instructors (n = 18, 32.7%), followed by interdisciplinary teams (n = 10, 18.2%) and licensed mental health clinicians (n = 9, 16.4%) (Fig 4C). Self-guided and externally facilitated formats were less common, and peer- or lived-experience facilitation was rare but emerging. Overall, faculty members, interdisciplinary teams, and licensed mental health clinicians most commonly delivered interventions, whereas peer- and lived-experience facilitation was reported less frequently.

As shown in Fig 4D, interventions commonly addressed multiple MHL domains. All included interventions addressed knowledge, attitudes/stigma, skills, confidence, and behavior change, whereas self-awareness and help-seeking were addressed slightly less frequently. Because individual interventions often targeted multiple domains, these categories were not mutually exclusive. Overall, the mapped interventions were predominantly short-term and faculty-led, with a strong emphasis on knowledge- and attitude-related outcomes, while comparatively fewer incorporated experiential, technology-enhanced, or co-produced educational approaches.

Technology integration was primarily achieved through e-learning platforms (12 studies), such as Moodle and edX (Table 1). Video materials were used in 10 studies, simulation tools (vSim, INMEDEA) in 8, and videoconferencing tools (e.g., Zoom) in 6. Interactive tools were the least common (4 studies). Only one peer-led program and one virtual simulation platform were identified, with no reports of AI or immersive virtual reality applications. Experiential learning strategies were prevalent, including role-play and simulation (15 studies), reflective exercises (10), real-world clinical contact (8), and mindfulness/self-reflection (5). Role-play/simulation was the most frequently reported experiential component, followed by reflective exercises, real-world clinical contact, and mindfulness/self-reflection (Table 1).

**Table 1:** Types of technology and experiential components used in mental health literacy educational interventions for health professions students, along with their frequency and illustrative examples. Percentages may exceed 100% because studies could be coded in multiple categories.

Technology Type	Category Explanation	*n (%)*
Simulation-based learning	Involves using digital or virtual environments that mimic clinical scenarios for hands-on training, such as virtual patient simulators (e.g., vSim, INMEDEA).	8 (14.5%)
Video / Film materials	Utilizes recorded audiovisual content for instruction, demonstration, or reflection (e.g., filmed patient encounters, instructional videos).	10 (18.2%)
Online / e-learning platforms	Refers to web-based learning management systems or structured online modules used for content delivery (e.g., Moodle, edX, EasyGenerator).	12 (21.8%)
Video conferencing tools	Employs synchronous online communication technologies to facilitate remote teaching, discussions, or assessments (e.g., Zoom, Microsoft Teams, Google Meet).	6 (10.9%)
Surveys / interactive tools	Involves digital tools enabling participation, feedback, or interactive learning (e.g., electronic surveys, polls, real-time quiz systems).	4 (7.3%)
***Experiential Component Type***		
Role-play / Simulation	Involves experiential learning through reenactment of clinical or interpersonal scenarios, often using standardized patients or simulated environments to develop communication and clinical decision-making skills.	15 (27.3%)
Reflective Exercises	Includes structured written or verbal reflections encouraging learners to analyze personal experiences, attitudes, and emotional responses to enhance self-awareness and empathy.	10 (18.2%)
Real-world / Clinical Contact	Incorporates direct patient engagement or clinical environments, allowing learners to apply theoretical knowledge in authentic healthcare settings.	8 (14.5%)
Mindfulness / Self-reflection	Focuses on activities that promote personal awareness, emotional regulation, and resilience through mindfulness practices or guided self-reflection exercises.	5 (9.1%)

Note: Percentages are based on the total number of included studies (n = 55). Percentages do not sum to 100% because individual studies could include multiple technology types and experiential components and were therefore counted in more than one category.

### Theoretical frameworks

In addition to differences in intervention design, studies varied in the theoretical frameworks that informed MHL curricula and educational delivery (S 1G in S1 File). The most common were Mental Health Literacy & Stigma frameworks, notably Jorm’s MHL framework [25–28]. Several studies also drew on the Mental Health First Aid (MHFA) program, including its ALGEE action plan (Approach, Listen, Give support, Encourage professional help, and Encourage other supports) [25,28]. Prominent psychological models included Cognitive Behavioral Therapy (CBT) [29,30], Motivational Interviewing (MI) [31], and Mindfulness-Based Stress Reduction (MBSR) [29,32]. Educational models such as Kern’s six-step approach [33] and the Knowledge-Attitude-Practice (KAP) model [34] were also used. Community and philosophical frameworks, such as Stoic Philosophy [35] and the Ladder of Involvement [36], were reported less frequently. Overall, MHL and stigma-related frameworks were the most commonly reported conceptual foundations, whereas psychological, educational, and community-based models were applied less consistently across interventions.

### Mental health literacy components

The diversity of intervention designs was reflected in the range of mental health literacy topics addressed across the included studies (S 1H in S1 File). The most frequently addressed topics were depression (n = 42), anxiety (n = 39), suicide prevention (n = 34), substance use disorders (n = 22), and psychosis (n = 18). Other themes included stress management, stigma reduction, and communication skills. Fewer studies focused on emerging areas like mindfulness (n = 10), family contexts (n = 8), and human rights (n = 6). Topics such as trauma (n = 5), eating disorders (n = 4), and maternal mental health (n = 3) were rarely targeted. Overall, the mapped interventions focused primarily on common mental disorders, whereas topics such as trauma, eating disorders, maternal mental health, and human rights were addressed less frequently.

### Evaluation and assessment

Variation was also evident in how educational outcomes were evaluated and the extent to which interventions demonstrated effects beyond immediate learning. Outcomes were predominantly mapped to Level 2 of the Kirkpatrick framework, specifically attitude (2a), knowledge (2b), and confidence (2c) (Fig 5). These domains were most frequently assessed in the Attitude & Stigma, Knowledge, and Mental Health & Well-being categories. As shown in Fig 5, only a small number of studies reported outcomes at Kirkpatrick Level 3 (behavior), and none assessed outcomes at Kirkpatrick Level 4 (organizational impact). Overall, most reported outcomes were mapped to Kirkpatrick Level 2, whereas relatively few studies evaluated Level 3 outcomes, and none assessed Level 4 outcomes.

**Fig 5:**
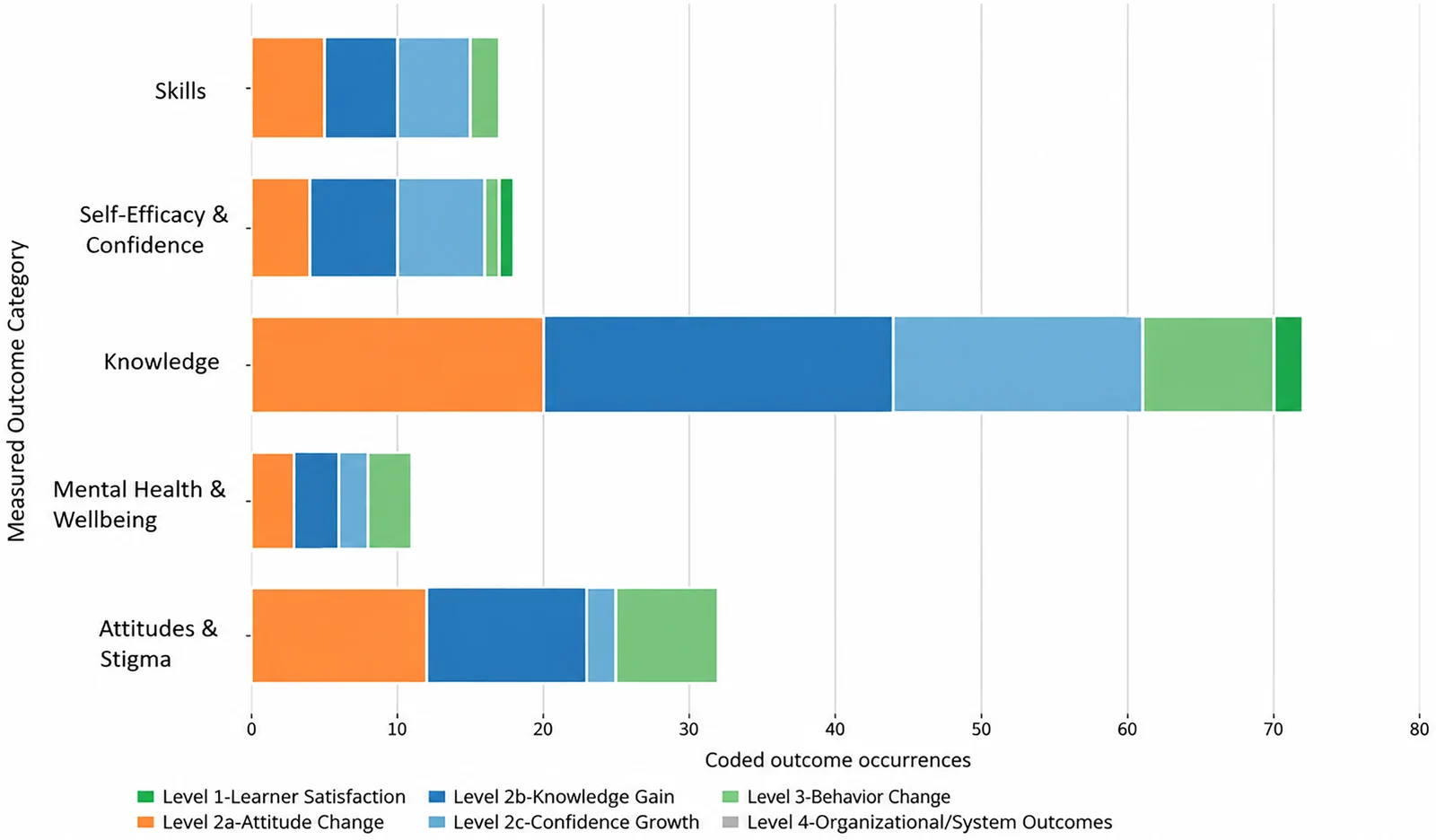
Distribution of measured outcome categories across Kirkpatrick evaluation levels (coded outcome occurrences). Categories were not mutually exclusive; individual studies could contribute to more than one outcome category and Kirkpatrick level..

A variety of validated instruments were used to assess outcomes related to MHL education across studies (S 1I in S1 File). Commonly used scales included the Opening Minds Stigma Scale (OMS-HC) and the Mental Illness Clinicians’ Attitudes Scale (MICA), measuring attitudes, stigma, and beliefs. Tools such as the Perceived Stress Scale (PSS-10) and the Depression, Anxiety, and Stress Scale (DASS-21) assess stress and well-being. However, assessments of help-seeking behaviors and suicide prevention competence were less frequent. In contrast, some studies measured self-efficacy, empathy, and communication skills. Very few examined reflection or professional growth. Overall, a wide range of instruments was used to assess cognitive and affective outcomes, whereas measures of behavioral change, professional practice, and long-term outcomes were reported less frequently. Evaluation schedules in the studies were primarily short-term (S 1J in S1 File). Among the six RCTs, five used a pre–post design, and only two included follow-up assessments within one to three months post-intervention; none extended beyond six months. Of 26 quasi-experimental studies, 20 conducted immediate pre–post evaluations, while six had delayed follow-ups, often within the same academic term. Overall, long-term follow-up assessments were uncommon across the included studies. Across the studies, self-assessment was the predominant evaluation method, with most interventions relying on student-reported gains in knowledge, confidence, and attitudes (S 1K in S1 File). Faculty-led or objective assessments were less common and typically limited to simulation or Objective Structured Clinical Examination (OSCE)-based designs. Only a small subset of studies incorporated peer evaluation. Overall, the mapped evaluations relied predominantly on short-term, self-reported learning outcomes, with comparatively few studies incorporating long-term follow-up, behavioral outcomes, or multi-source assessment approaches.

### Implementation and contextual factors

Beyond educational outcomes, several studies reported contextual factors that influenced the implementation and feasibility of mental health education interventions, grouped into three interrelated domains: cultural, institutional, and logistical (Table 2). Culturally, stigma rooted in religious beliefs, particularly in Nigeria, views mental illness as a punishment or possession, leading to reliance on traditional healers [37]. Community engagement and the involvement of religious leaders were described as facilitating implementation, with support from culturally adapted training approaches and increased openness to mental health during the pandemic. Institutionally, barriers included outdated legislation like Nigeria’s Lunacy Act (1958), inadequate funding, and limited integration of mental health into curricula. Facilitators included policy reforms, government subsidies, and structured international collaborations. Logistical challenges, including shortages of mental health professionals and high treatment costs, were commonly reported. These challenges were addressed through free or low-cost online delivery formats, coordinated institutional support, and enhanced accessibility via digital and community-based initiatives [29,30]. Overall, implementation was influenced by interacting cultural, institutional, and logistical factors, with both barriers and facilitators reported across the included qualitative studies.

**Table 2:** Contextual barriers and facilitators to mental health education and engagement identified in included qualitative studies.

Domain	Barriers	Facilitators
**Cultural**	**Stigma and beliefs** Mental illness is often viewed as a spiritual attack, punishment, or possession. **Traditional reliance** Preference for religious/traditional healers before professional care. **Cultural resistance** Negative societal attitudes (e.g., in Nordic contexts) and stigma surrounding help-seeking.	**Community engagement** Educating religious/community leaders to guide people to appropriate care. **Cultural sensitivity** Adapting training to local norms (e.g., including family, cultural expressions like “Allah’s will”). **Pandemic influence** Increased openness and reduced stigma around mental health. **International diversity** Exposure to diverse cultural perspectives has enhanced our understanding of mental health globally.
**Institutional**	**Outdated policies** Old mental health laws (e.g., Lunacy Act 1958, an outdated statute recently replaced in Nigeria), or limited policy implementation. **Curriculum gaps** Lack of mental health education, counseling services, and self-help training in universities. **Limited institutional support** Consumer (Expert by Experience) participation in education is often “ad hoc” and unsustained. **Accessibility issues** Long waiting times, bureaucratic barriers to psychological support.	**Government and institutional reforms** Moves toward anti-discrimination policies and integration of mental health into primary care. **Strong institutional partnerships** Funded, formal international projects supporting Expert by Experience involvement. **Supportive structures** Institutional mediation for recruitment; funding from nursing schools or health agencies. **Educational flexibility** Acceptance of simulation-based learning replacing clinical hours.
**Logistical**	**Access and cost** Scarcity of treatment centers (primarily in rural areas), high costs, and a shortage of professionals. **Scheduling and workload** Sessions clashing with academic/work schedules; poor timing (e.g., final semester). **Structure and participation** Disjointed session design, weak integration, wiki participation, and group dynamics issues. **Limited adaptation** Training is not fully tailored to the student’s specific needs.	**Accessibility** Remote, low-cost, and flexible delivery (online/regular classrooms). **Supportive logistics** Free for students, institutional funding, and simulated materials (patient charts). **Mass communication** Use of media and social platforms to improve awareness and access to care. **International coordination** Well-organized multi-university collaboration and project management.

## Discussion

By systematically mapping the global evidence base, this evidence and gap mapping study provides the first comprehensive overview of how mental health literacy education for health professions students has been designed, implemented, and evaluated across contexts. Although research activity has increased substantially since 2016, the evidence base remains concentrated within a limited number of regions, professions, and educational approaches. The predominance of short-term, quasi-experimental studies and faculty-led instructional models reflects a field that remains in an early stage of educational development, with limited evidence supporting sustained, curriculum-integrated approaches. Similarly, the relatively limited use of experiential, co-produced, and technology-enhanced approaches indicates that innovation has not yet become a defining characteristic of the field. Collectively, these findings suggest that while MHL education is increasingly recognized as an important component of health professions training, its development remains uneven and has not yet fully translated into sustainable, comprehensive, and globally representative educational practice. In addition, the marked increase in publications after 2016 likely reflects the combined influence of growing policy attention to mental health and workforce resilience [38–40], the heightened emphasis on mental health following the COVID-19 pandemic [41], and the rapid expansion of digital and blended learning approaches within health professions education [42–44].

From an educational perspective, the predominance of short-term, faculty-led, and didactic interventions suggests that MHL education remains primarily focused on knowledge transmission rather than on competency development. Although an increasing number of studies incorporated simulation, role-play, reflective learning, and structured contact with individuals with lived experience, these approaches remained comparatively uncommon despite growing evidence supporting their role in reducing stigma, strengthening empathy, and improving communication skills [16,45,46].This pattern suggests that future curricula should move beyond isolated teaching sessions toward longitudinal, experiential learning models that better prepare students for the complexities of mental healthcare.

A second notable finding of this evidence map is the marked geographical imbalance in the published literature. More than half of the included studies originated from Europe and the Western Pacific Region, whereas the African and Eastern Mediterranean regions contributed relatively few studies, and no eligible studies were identified from the WHO South-East Asia Region. Although this distribution mirrors broader trends in global mental health research [47], it raises important questions about the generalisability of the current evidence base. Educational interventions developed and evaluated in high-income settings may not be readily transferable to low- and middle-income countries, where mental health systems, educational infrastructure, cultural beliefs, and workforce capacity differ substantially [38]. Expanding research in underrepresented regions should therefore involve more than increasing publication output; it should prioritize the development and evaluation of culturally responsive, contextually adapted MHL educational models that reflect local priorities, healthcare systems, and sociocultural understandings of mental health [48].

From a methodological perspective, the evidence base remains dominated by evaluations of immediate learning outcomes, with most studies assessing changes in knowledge, attitudes, and confidence corresponding to Kirkpatrick Level 2. In contrast, relatively few studies examined behavioral change (Level 3), and none evaluated organizational or system-level outcomes (Level 4). This pattern suggests that although existing interventions appear successful in improving proximal educational outcomes, there is limited evidence on whether these improvements translate into sustained changes in clinical practice or in broader educational and healthcare systems. Similar concerns have been raised in previous research, highlighting reliance on short-term, self-reported measures and a lack of standardized assessment approaches in MHL education [13,49]. Future research should therefore prioritize longitudinal evaluation designs, objective assessments of behavioral change, and implementation outcomes that better capture the real-world impact of MHL education on professional practice.

This evidence and gap mapping study extends the existing literature by moving beyond questions of intervention effectiveness to examine how MHL education has been designed, implemented, and evaluated across different educational and geographical contexts. Unlike previous systematic and scoping reviews that primarily synthesized intervention outcomes, this review integrates evidence on pedagogical approaches, theoretical frameworks, implementation factors, and evaluation strategies within a single analytical framework. By identifying both evidence clusters and persistent research gaps, this review offers a structured overview of where evidence is currently concentrated and where important knowledge gaps remain. This broader perspective may assist educators, researchers, and policymakers in prioritizing future curriculum development, implementation strategies, and research investment in mental health literacy education.

### Educational, policy, and research implications

The following implications are derived from the patterns and gaps identified in the evidence map and should be interpreted as directions for future research, policy, and practice rather than conclusions about intervention effectiveness.

From an educational perspective, future MHL initiatives should move beyond isolated, didactic instruction toward integrated, learner-centered approaches that span multiple stages of professional training. Curriculum designers must prioritize experiential and reflective strategies, including simulation, case-based learning, real-world clinical contact, and co-produced learning with individuals with lived experience. Embedding MHL longitudinally within curricula, alongside peer-led and interprofessional learning models, may support competency development, reflective practice, collaboration, and empathy, in alignment with adult learning principles.

At the policy level, sustained investment is required to strengthen capacity for MHL education, particularly in low- and middle-income countries. Policymakers and institutional leaders must support faculty development, curricular integration, and standardized reporting frameworks to promote equity, scalability, and sustainability. Global policy instruments, including the WHO Mental Health Action Plan (2013–2030) [40] and Sustainable Development Goal 3.4 [39], can be leveraged by accreditation and professional bodies to mandate core MHL competencies across health professions programs. Cross-sector collaboration and systematic inclusion of lived-experience perspectives are essential to support system-wide adoption.

From a research standpoint, future studies must prioritize longitudinal and randomized designs capable of assessing behavioral, clinical, and organizational outcomes (Kirkpatrick Levels 3–4). Researchers must improve transparency in sampling, intervention fidelity, implementation reporting, and incorporate economic and feasibility evaluations to inform sustainable scale-up. Development and validation of assessment instruments specific to health professions students, alongside greater use of multi-source outcome measures, would strengthen methodological rigor. Emerging digital approaches, including virtual reality, artificial intelligence, and adaptive e-learning systems, represent important areas for future investigation.

### Strengths and limitations

A key strength of this EGM study lies in its broad scope, structured synthesis, and visual evidence-mapping approach, which together provide an overview of 55 studies across multiple professions, countries, and intervention types. Use of the EGM framework enabled identification of thematic clusters, geographic disparities, and critical research gaps, offering a valuable reference for educators and policymakers. However, several limitations must be noted. Restricting publications to English-language sources may underrepresent evidence from non-Anglophone regions, particularly from low- and middle-income countries. In addition, the mapping approach prioritizes breadth over methodological rigor, necessitating cautious interpretation of mapped intervention characteristics and reported outcomes. Finally, despite extensive database coverage, some grey literature and unpublished evaluations may have been missed, potentially leading to an underestimation of local innovation. Future mapping efforts must incorporate multilingual searches and systematic screening of grey literature to improve inclusivity and representativeness.

## Conclusion

This Evidence and Gap Mapping (EGM) scoping review demonstrates that MHL education for health professions students has expanded substantially over the past decade but remains geographically uneven and methodologically fragmented. Most interventions are short-term, faculty-led, and didactic, with limited integration of experiential, co-produced, or technology-enhanced learning. While evidence of educational outcomes is growing, few studies have examined long-term behavioral or systemic impacts. To address identified gaps, future efforts must prioritize longitudinal, theory-informed, and contextually adaptable designs that embed reflective practice, interdisciplinary collaboration, and involvement of lived experiences. Stronger policy alignment, investment in LMICs, and the use of innovative digital tools such as virtual and augmented reality can further enhance reach and sustainability. By addressing these gaps, MHL education can evolve into an equitable, scalable, and integral component of global health professions training, thus empowering future practitioners to promote mental well-being within their communities and clinical practice.

## Supporting information

S1 File Summary of data assessment. (DOCX)

S2 File Underlying study dataset. (PDF)
