# Supplementary material for: Mental health literacy training interventions for future health professionals: An evidence and gap mapping scoping review
Source: PLoS One. 2026 Oct 27;21(10):e0360403. doi: 10.1371/journal.pone.0360403 (PMC13712762; doi:10.1371/journal.pone.0360403)
Supplement: Supplementary file 1 [file pone.0360403.s002.pdf]

Study ID	DOI / URL	Author(s) et al.	Year	Journal	Country	WHO Region	Setting	Study Context (Single / Multi-site)	Study Design	Health Profession(s)	Professional Composition	Education Level	Sample (n)	Sampling Method	Intervention Name	Type of Intervention	Duration	Mode of delivery
QU01	[illegible]	Olumujiba O. Bamgbose, Adeninke Bamgbose Pederson, Anuneweza A. O. Okocha	2023	International Journal for the Advancement of Counselling	Nigeria	African Region (AFR)	University classroom	Single	Qualitative descriptive study	Nursing students, Pharmacy students, Medical students	Multi-professional	Undergraduate, Postgraduate	40	Purposive	Mental Health Awareness Seminar + Focus Groups	Seminar	Very short-term	Face-to-face
QU02	[illegible]	Chan YY, Ting CH	2023	Journal of Postgraduate Medicine	Malaysia	Western Pacific Region (WPR)	University classroom	Single	Qualitative descriptive study	Medical students	Uniprofessional	Undergraduate	305	Convenience	Psychological Skills Lab (PSL)	Simulation-based	Extended/Continuous	Blended
QU03	[illegible]	[illegible]	2023	Issues in Mental Health Nursing	Brazil	Region of the Americas (AMR)	Online environment	Two-site	Qualitative descriptive study	Nursing students	Uniprofessional	Undergraduate students	20	Purposive	Online Psychoeducation Group (8 weeks)	Online / E-learning Modules	Medium-term	Online/e-learning
QU04	[illegible]	[illegible]	2019	Counselling and Psychotherapy Research	Australia, Iceland, Ireland, Finland, the Netherlands, Norway	Western Pacific Region (WPR)	University classroom	Multi-site (International)	Qualitative exploratory study	Nursing students	Uniprofessional	Undergraduate	51	Purposive	Expert-by-Experience (EBE) Co-Produced Module	Contact-based Education	Extended/Continuous	Face-to-face
QU05	[illegible]	[illegible]	2019	Counselling and Psychotherapy Research	Australia, Iceland, Ireland, Finland, the Netherlands, Norway	Western Pacific Region (WPR)	University classroom	Multi-site (International)	Qualitative exploratory study	Nursing students	Uniprofessional	Undergraduate	51	Purposive	Co-Produced Learning Module (EBE-Led)	Contact-based Education	Extended/Continuous	Face-to-face
QU06	[illegible]	[illegible]	2019	Collegian	Hong Kong	Western Pacific Region (WPR)	University classroom	Single	Qualitative descriptive study	Nursing students	Uniprofessional	Undergraduate	25	Purposive	Mental Health First Aid (12-hour course)	workshop/training	Very short-term	Face-to-face
QU07	[illegible]	Sheridan, Rhonda; Williams, Jacqui	2022	Collegian	USA	Region of the Americas (AMR)	University classroom	Single	Qualitative descriptive study	Nursing students	Uniprofessional	Undergraduate	[illegible]	Convenience	Cinematic Simulation	Simulation-based	Very short-term	Face-to-face
QU08	[illegible]	Sibeoni J, Ellul P, Bubola T, Dadache Y, Flori M A	2023	Collegian	France	European Region (EUR)	University classroom	Single	Qualitative phenomenological study	Nursing students, Pharmacy students, Medical students, Physical Therapy students, Dentistry students	Multi-professional	Undergraduate	20	Purposive	Mental Health First Aid (2-day French version)	workshop/training	Very short-term	Face-to-face
QU09	[illegible]	[illegible]	2013	Collegian	United Kingdom	European Region (EUR)	University classroom	Single	Qualitative descriptive study	Nursing students, Social work students, Clinical Psychology students, Psychiatry Residents	Multi-professional	Undergraduate, postgraduate	130	Convenience	Collaborative Learning Initiative (CLI)	Contact-based Education	Very short-term	Blended
QU10 (MMO3)	[illegible]	Jenny Kelly, Melanie Birks	2017	Collegian (2017)	Australia	Western Pacific Region (WPR)	University classroom	Single	Qualitative	Nursing students, Midwifery students	Multi-professional	Undergraduates	66	Convenience	Tailored 13-hour Mental Health First Aid Course	workshop/training	Very short-term	Face-to-face
MM01	[illegible]	[illegible]	2022	BMC Medical Education (2022)	United Kingdom	European Region (EUR)	Online environment	Single	Mixed method , Convergent parallel	Medical Students	Uniprofessional	Undergraduates	24	Convenience	SelfenE – Stoic Reflection for Resilience and Empathy	Online / E-learning Modules	Short-term	Online/e-learning
MM02	[illegible]	[illegible]	2018	Academic Pediatrics (2018)	United States	Region of the Americas (AMR)	University classroom	Two-site	Mixed-Methods ,Convergent parallel design	Pediatricians, Residents, Psychology Residents, Medical students, Social work students	Multi-professional	Residents, Interns	50	Convenience	Integrated Mental Health Training Curriculum using SP encounters	Contact-based Education	Short-term (repeated each semester)	Face-to-face
MM04	[illegible]	[illegible]	2016	Nurse Education in Practice (2016)	Scotland, United Kingdom	European Region (EUR)	University classroom	Two-site	Mixed-method,Sequential explanatory	Nursing students, Occupational Therapy students	Multi-professional	Undergraduates	143	Convenience	Alcohol Brief Intervention (ABI) Workshop	workshop/training	Very short-term	Face-to-face
MM05	[illegible]	[illegible]	2023	American Journal of Pharmaceutical Education (2023)	United States	Region of the Americas (AMR)	University classroom	Single	Mixed-Methods,Convergent parallel	Pharmacy students	Uniprofessional	Undergraduates	202	Convenience	Neuropsychiatric Therapeutics + Case-Based Course	Case-based Education	Extended/Continuous	Face-to-face
MM06	[illegible]	Syed Sheriff RJ, Bass N, Hughes P, Ade-Odunlade P, Ismail A, Whitwell S, Jenkins R	2013	African Journal of Psychiatry (2013)	Somaliland	African Region (AFR)	University classroom	Two-site	Mixed-Methods,Convergent parallel design	Medical students	Uniprofessional	Undergraduates	35	Census sampling	Interactive Psychiatry Training Program (7-day course)	workshop/training	Very short-term	Face-to-face
OB01	No psychiatry? Assessment of family medicine residents' training in mental health issues	Client Bethune, Graham Worrall, Duron Freake, Elizabeth Church	1999	Canadian Family Physician (1999)	Canada	Region of the Americas (AMR)	Clinical rotation	Single	Cross-sectional	Family Physicians Residents	Uniprofessional	Postgraduate	62	Census sampling	Integrated multidisciplinary behavioral medicine curriculum	Integrated Curriculum Course	Extended/Continuous	Face-to-face
OB02	[illegible]	[illegible]	2021	Nurse Education Today (2022)	Italy	European Region (EUR)	University classroom	Multi-site	Cross-sectional	Nursing students	Uniprofessional	Undergraduates	314	Convenience	Suicide Risk Lecture (2-hour specific class)	lecture-based	Very short-term	Face-to-face
OB03	[illegible]	[illegible]	2022	PLOS ONE (2022)	Germany	European Region (EUR)	University classroom	Single	Cross-sectional	Medical students	Uniprofessional	Undergraduate	[illegible]	Convenience	Digital Mental Health Curriculum (Kern's six-step)	Integrated Curriculum Course	Extended/Continuous	Blended
OB04	[illegible]	Wei Liu	2021	Issues in Mental Health Nursing (2021)	United States	Region of the Americas (AMR)	Online environment	Single	Prospective Cohort	Nursing students	Uniprofessional	Undergraduate	[illegible]	Convenience	Virtual Simulation Training in Mental Health Nursing	Virtual simulation	Extended/Continuous	Online/e-learning
OB05	[illegible]	[illegible]	2021	Nurse Educator (2021)	United States and Turkey	Region of the Americas (AMR)	Simulation lab	Multi-site	Cross-sectional	Nursing students	Uniprofessional	Undergraduate	[illegible]	Convenience	Simulated Patient (SP) Mental Health Scenarios	Simulation-based	Medium-term	Face-to-face
OB06	[illegible]	[illegible]	2022	International Journal of Environmental Research and Public Health (2022)	Spain	European Region (EUR)	University classroom, Clinical rotation	Two-site	Prospective Cohort	Nursing students	Uniprofessional	Undergraduate	162	Convenience	Integrated Mental Health Training and Clinical Practice	Integrated Curriculum Course	Extended/Continuous	Face-to-face
OB07	[illegible]	[illegible]	2017	Prevention Science (2017)	United Kingdom	European Region (EUR)	University classroom	Multi-site	Cross-sectional	Medical students	Uniprofessional	Undergraduate	[illegible]	Convenience	OpenMinds Peer-Led Programme	Contact-based Education	Short-term	Face-to-face
OB08	[illegible]	[illegible]	2015	Education for Health (2015)	Australia	Western Pacific Region (WPR)	University classroom	Single	Cross-sectional	Medical students, Paramedical students, Pharmacy students	Multi-professional	Undergraduate	288 students (102 Medical, 113 Paramedical, 51 Pharmacy)	Census sampling	Suicide Awareness and Intervention Program (SAIP)	workshop/training	Very short-term	Face-to-face
RCT01	[illegible]	[illegible]	2023	European Child & Adolescent Psychiatry (2023)	Australia	Western Pacific Region (WPR)	University classroom	Single	RCT	Dentistry students, Nursing students, Psychology students, Occupational Therapy students, Social work students	Multi-professional	Undergraduate	[illegible]	Convenience	Talk-to-Me MOOC – Suicide Prevention & Mental Health Education	Online / E-learning Modules	Medium-term	Online/e-learning
RCT02	[illegible]	Sevkut Bahar-Özvaris, Füsun Cuhadaroglu Çetin, Sevgi Turan, Antoinette S. Peters	2006	Medical Teacher (2006)	Turkey	European Region (EUR)	University classroom	Single	RCT	Medical students	Uniprofessional	Undergraduate	Fifth-year undergraduate medical	Convenience	Cooperative Learning PBL in Mental Health	Problem-based Education ,Team-based Education	Medium-term	Face-to-face
RCT03	[illegible]	[illegible]	2017	BMC Psychiatry (2017)	Australia	Western Pacific Region (WPR)	University classroom	Single	RCT	Nursing students	Uniprofessional	Undergraduate	[illegible]	Convenience	Standardized MHFA Course for Nursing Students	workshop/training	Very short-term	Face-to-face
RCT05	[illegible]	[illegible]	2018	BMC Medical Education (2018)	United Kingdom	European Region (EUR)	University classroom	Single	RCT	Medical students	Uniprofessional	Undergraduate,Postgraduate	[illegible]	Purposive	MHFA eLearning Course (6-week online)	Integrated Curriculum Course	Medium-term	Online/e-learning
RCT06	[illegible]	[illegible]	2013	BMC Medical Education (2013)	Norway	European Region (EUR)	University classroom	Two-site	RCT	Medical students and Psychology students	Multi-professional	Undergraduate	[illegible]	Convenience	Mindfulness-Based Stress Reduction (7-week)	workshop/training	Medium-term	Face-to-face
RCT07	[illegible]	[illegible]	2016	Academic Psychiatry (2016)	Malaysia	Western Pacific Region (WPR)	University classroom	Single	RCT	Medical students	Uniprofessional	Undergraduate	[illegible]	Convenience	Brief Psychoeducation + Contact (Face-to-Face vs Video)	lecture-based	Very short-term	Face-to-face
QS01	[illegible]	Susan Edgar, Joanne Connaughton	2021	Physiotherapy Canada (2021)	Australia	Western Pacific Region (WPR)	University classroom	Single	Quasi-experimental	Physical Therapy students	Uniprofessional	Undergraduate	[illegible]	Convenience	Mental Health First Aid (MHFA) Training	workshop/training	Unspecified (not reported)	Face-to-face

Study ID	Facilitators	Key mental health literacy topics covered	Study Aim / Focus	MHL_Domain_Knowledge(Y/N)	MHL_Domain_Attitudes_Stigma(Y/N)	MHL_Domain_Skills(Y/N)	MHL_Domain_HelpSeeking(Y/N)	MHL_Domain_SelfAwareness(Y/N)	MHL_Domain_Confidence(Y/N)	MHL_Domain_BehaviourChange(Y/N)
QU01	Interdisciplinary Team (Mixed roles)	[illegible]	Y	Y	Y	Y	Y	Y	Y	Y
QU02	Faculty / Instructor	[illegible]	Y	Y	Y	Y	Y	Y	Y	Y
QU03	Interdisciplinary Team (Mixed roles)	[illegible]	Y	Y	Y	Y	Y	Y	Y	Y
QU04	Lived-experience Co-facilitator	[illegible]	Y	Y	Y	Y	Y	Y	Y	Y
QU05	Lived-experience Co-facilitator	[illegible]	Y	Y	Y	Y	Y	Y	Y	Y
QU06	Not reported / Unknown	[illegible]	Y	Y	Y	Y	Y	Y	Y	Y
QU07	Faculty / Instructor		Y	Y	Y	Y	Y	Y	Y	Y
QU08	Interdisciplinary Team (Mixed roles)	[illegible]	Y	Y	Y	Y	Y	Y	Y	Y
QU09	Lived-experience Co-facilitator	[illegible]	Y	Y	Y	Y	Y	Y	Y	Y
QU10 (MMO3)	Not reported / Unknown	[illegible]	[illegible]	Y	Y	Y	Y	Y	Y	Y
MM01	Self-guided / No human facilitator	[illegible]	[illegible]	Y	Y	Y	N	Y	Y	Y
MM02	Licensed Mental Health Clinician	[illegible]	[illegible]	Y	Y	Y	Y	Y	Y	Y
MM04	External Trainer / Consultant	[illegible] structured ABI conversation.	[illegible] on knowledge & skills	Y	Y	Y	Y	N	Y	Y
MM05	Faculty / Instructor	Mental illness stigma, recovery, empowerment, empathy, communication	To evaluate stigma reduction following neuropsychiatric & case-based courses.	Y	Y	Y	N	Y	Y	Y
MM06	Interdisciplinary Team (Mixed roles)	Physical/Mental health overlap, communication skills (breaking bad news), classification, history taking, affective disorders, management.	To pilot interactive mental health training in a resource-poor setting.	Y	Y	Y	N	Y	Y	Y
OB01	Licensed Mental Health Clinician	Death and dying, depression, giving bad news, loss/bereavement, violence (wife assault, sexual assault), anxiety, addictions, crisis intervention, certification, parenting, marital problems, somatization. *Noted weaknesses:* ADHD, learning disorders, schizophrenia, child sexual abuse, adolescent suicide, marital therapy	To assess adequacy of integrated mental health training in family medicine residency.	Y	Y	Y	Y	Y	Y	Y
OB02	Faculty / Instructor	Suicide, suicidal risk, suicide care, and the nursing role. (Specific topics within this were not listed).	To assess nursing students' attitudes toward suicide and impact of education.	Y	Y	Y	Y	Y	Y	Y
OB03	Faculty / Instructor	Anxiety disorders, depression, trauma-related disorders, eating disorders, somatic symptom disorders, motivational interviewing. |	To evaluate effectiveness of an online mental health teaching concept during COVID-19.	Y	Y	Y	Y	Y	Y	Y
OB04	Self-guided / No human facilitator	Management of five specific mental disorders: severe anxiety, alcohol withdrawal, bipolar disorder, major depression, and schizophrenia. |	To evaluate long-term effects of virtual simulation on mental health literacy.	Y	Y	Y	Y	Y	Y	Y
OB05	Not reported / Unknown	Mental health examination, therapeutic communication, safety checks, patient teaching for Major Depressive Disorder and Schizophrenia. Cultural competence was integrated by tailoring patient backgrounds (religion, immigrant status). |	To evaluate diverse mental health simulation on learning and self-confidence.	Y	Y	Y	Y	Y	Y	Y
OB06	Not reported / Unknown	Not explicitly detailed in the article. The focus of the study was on the impact of the overall curriculum on stigma, not its specific content. |	To examine changes in stigma and behavior in nursing students during mental health training	Y	Y	Y	Y	Y	Y	Y
OB07	Interdisciplinary Team (Mixed roles)	Depressive disorders, anxiety disorders, psychosis, eating disorders, substance misuse, self-harm, help-seeking, reducing stigma	To evaluate efficacy and acceptability of a peer-led mental health literacy program.	Y	Y	Y	Y	Y	Y	Y
OB08	Professional Staff (Non-clinical)	Suicide myths & statistics, the "suicide journey" (using analogies), risk assessment (ABC model: Ask, Behaviours, Current plan, Discover), communication skills, available support services. |	To assess impact of suicide awareness training for health professional	Y	Y	Y	Y	Y	Y	Y
RCT01	Self-guided / No human facilitator	Mental fitness, self-injury, suicidal behavior, gatekeeper interventions	Evaluate the efficacy of the "Talk-to-Me" MOOC suicide prevention program for tertiary	Y	Y	Y	Y	Y	Y	Y
RCT02	Faculty / Instructor	Adolescent Development and Psychopathology	Test cooperative PBL vs lecture in mental health training.	Y	Y	Y	N	Y	Y	Y
RCT03	External Trainer / Consultant	Recognition of mental health problems (depression, anxiety, substance use, psychosis, eating disorders), knowledge of mental health issues, crisis intervention strategies, reducing stigma. |	Evaluate MHFA training effects on literacy, stigma, and confidence.	Y	Y	Y	Y	Y	Y	Y
RCT05	Self-guided / No human facilitator	Definitions, prevalence, risk factors, interventions, and crisis management for: Depression, Anxiety, Eating disorders, Psychosis, Substance use problems. The MHFA 'ALGEE' action plan.	Assess MHFA eLearning's impact on literacy & stigma.	Y	Y	Y	Y	Y	Y	Y
RCT06	Faculty / Instructor	Stress management, mindful communication, cultivating an "open, curious, non-judgemental and accepting attitude". Focus on coping skills and self-awareness rather than specific mental illness education.	Evaluate mindfulness training for stress & well-being.	Y	Y	Y	Y	Y	Y	Y
RCT07	Faculty / Instructor	Prevalence of mental illness, biological and psychosocial models, impacts of mental illness, etiopathogenesis of stigma, effects of stigma, recovery-oriented paradigm, myth-busting. |	Examine brief psychoeducation effects on stigma in medical students.	Y	Y	Y	Y	Y	Y	Y
QS01	Not reported / Unknown	| Recognition of signs/symptoms, communication strategies, crisis response, reducing stigma, asking about suicidal ideation. |	Assess MHFA training effects on physiotherapy students' literacy, attitudes, and confidence	Y	Y	Y	Y	Y	Y	Y

Study ID	Experiential Component (Y/N)	Experiential Component Type (Unified)	Technology Type (Unified)	Instructional Components (Unified)	Assessment / Evaluation Method	Timing of Evaluation	Assessor of Intervention	Measured Outcomes categories	Assessment Instrument(s)	Kirkpatrik level	Key Findings
QU01	Y	Not reported	Video / Film materials	Didactic teaching / Lectures; Video / multimedia	Focus groups + thematic analysis	Post	Mixed evaluation (faculty & researcher)	Attitudes & Stigma	interviews	Level 2a, Level 2b	Positive: Students identified education as core to reducing stigma; supported social contact & counseling access.
QU02	Y	Role-play / Simulation; Reflective Exercises	Online / e-learning platforms; Video / Film materials	Didactic teaching / Lectures; Group discussions / Peer learning; Role-play / Simulation; Case-based learning; Mindfulness / Reflective activities	Narrative descriptive qualitative evaluation	Ongoing (curriculum-based)	Faculty-led evaluation	Skills	Observation/Reflection tool	Level 2a, Level 2b, Level 2c	Positive: PSL improved self-awareness, empathy & peer support; promoted autonomy & resilience.
QU03	Y	Reflective Exercises	Video conferencing tools; Surveys / interactive tools	Psychoeducation / Communication training; Mindfulness / Reflective activities	Focus groups + COREQ-guided analysis	Post	Researcher-led evaluation	Knowledge	interviews	Level 2a, Level 3	Positive: Promoted sharing, support networks & mental health awareness; reduced isolation.
QU04	Y	Real-world / Clinical Contact; Reflective Exercises	Not reported	Patient / Service user involvement	Focus groups + thematic analysis	Post	Mixed evaluation (faculty & researcher)	Attitudes & Stigma	interviews	Level 2a, Level 2c	Positive: Highlighted value of lived experience & recovery model; students advocated structured EBE involvement.
QU05	Y	Real-world / Clinical Contact	Not reported	Patient / Service user involvement	Qualitative focus groups (thematic)	Post	Mixed evaluation (faculty & researcher)	Knowledge	interviews	Level 2a, Level 2b, Level 2c	Positive: Students gained deeper empathy; recognized 'person beyond diagnosis'; reinforced recovery practice.
QU06	Y	Real-world / Clinical Contact	Not reported	Psychoeducation / Communication training	Semi-structured interviews	Post	Mixed evaluation (faculty & researcher)	Knowledge	interviews	Level 2a, Level 2b, Level 2c	Positive: MHFA enhanced literacy, confidence, skills, & self-awareness; students valued experiential approach.
QU07	Y	Role-play / Simulation	Video / Film materials; Simulation-based learning	Role-play / Simulation; Video / multimedia; Assessment / Feedback activities	Reflections + qualitative feedback	Post	Faculty-led evaluation	Self-Efficacy & Confidence	Observation/Reflection tool	Level 2b, Level 2c	Positive: Enhanced confidence, decreased fear; cinematic simulation effective & engaging for PMH nursing.
QU08	Y	Role-play / Simulation	Video / Film materials	Didactic teaching / Lectures; Video / multimedia; Role-play / Simulation; Group discussions / Peer learning	Semi-structured interviews (IPSE method)	Post	Researcher-led evaluation	Self-Efficacy & Confidence	interviews	Level 2a, Level 2b, Level 2c	Positive: Reported personal, professional & academic growth; promoted empathy & support skills.
QU09	Y	Real-world / Clinical Contact; Reflective Exercises	Online / e-learning platforms	Group discussions / Peer learning; Case-based learning; Video / multimedia	Post-course evaluations + thematic analysis	Post	Faculty-led evaluation	Attitudes & Stigma	interviews	Level 2b, Level 2c, Level 3	Positive: Effective interprofessional model; improved teamwork, understanding of roles, and attitudes.
QU10 (MMO3)	Y	Reflective Exercises	Online / e-learning platforms	Didactic teaching / Lectures; Video / multimedia; Role-play / Simulation; Group discussions / Peer learning	Online survey (post-course reflection)	Post	Student self-assessment	Knowledge	Developed survey/questionnaire	Level 1, Level 2a	Positive: 89% would recommend course; increased mental health literacy & confidence.
MM01	Y	Role-play / Simulation; Reflective Exercises	Video / Film materials	Mindfulness / Reflective activities	Mixed-methods: Pre/post psychometric scales (SABS, BRS, JSE) + interviews	Baseline, post, follow-up	Student self-assessment	Skills	Brief Resilience Scale; Jefferson Scale of Empathy; Self-Appraisal Behavioral Scale	Level 2a, Level 2b, Level 2c	Positive: Increased empathy & resilience; reflective Stoic mindfulness improved self-regulation.
MM02	Y	Role-play / Simulation	Video / Film materials	Didactic teaching / Lectures; Video / multimedia; Role-play / Simulation	Pre/post surveys + focus groups	Baseline, post, follow-up	Student self-assessment	Skills	Developed survey/questionnaire, Focus group	Level 2c, Level 3	Positive: Improved interdisciplinary confidence & collaboration; 96% applied warm handoffs post-training.
MM04	Y	Role-play / Simulation	Surveys / interactive tools	Didactic teaching / Lectures; Assessment / Feedback activities; Workshops / Interactive sessions; Video / multimedia	Pre/post/follow-up questionnaires + focus groups	Baseline, post,follow-up	Student self-assessment	Knowledge	Developed survey/questionnaire	Level 2b, Level 2c, Level 3	Positive: Knowledge & confidence doubled; participants applied ABI techniques in practice.
MM05	Y	Reflective Exercises	Online / e-learning platforms; Surveys / interactive tools	Didactic teaching / Lectures; Case-based learning	Pre/post/follow-up surveys	Baseline, post, follow-up	Student self-assessment	Skills	Opening Minds Stigma Scale for HealthCare Providers (OMS-HC), Recovery Scale (3-item version), Empowerment Scale (3-item version), Attribution	Level 2a, Level 2b	Positive: Significant reduction in stigma; improved empowerment & recovery attitudes.
MM06	Y	Role-play / Simulation; Real-world / Clinical Contact	Not reported	Didactic teaching / Lectures; Group discussions / Peer learning; Role-play / Simulation; Patient / Service user involvement	Pre/post MCQ, OSCE, attitude questionnaire	Baseline, post	Faculty-led evaluation	Knowledge	Exam/Performance-based assessment,Developed survey/questionnaire	Level 2b, Level 2c	Positive: MCQ improved from 50%→64%; OSCE avg 71%; reduced stigma, increased understanding.
OB01	Y	Real-world / Clinical Contact	Not reported	Clinical placements / Practical experience; Psychoeducation / Communication training; Didactic teaching / Lectures	Cross-sectional mailed survey	Post	Mixed evaluation (faculty & researcher)	Self-Efficacy & Confidence	Developed survey/questionnaire	Level 2b, Level 3	Positive: Graduates felt well-prepared; confidence linked to reduced referrals; program effective for mental health training.
OB02	Y	Not reported	Not reported	Unspecified / Not clearly detailed	Pre/post Suicide Behaviour Attitude Questionnaire (SBAQ-ita)	Baseline, follow-up	Researcher-led evaluation	Knowledge	Suicidal Behavior Attitude Questionnaire – Italian Version	Level 2a, Level 2b	Positive: Improved professional ability and reduced stigma; supports integrating suicide training into nursing curricula.
OB03	Y	Role-play / Simulation	Simulation-based learning; Online / e-learning platforms; Video / Film materials; Video conferencing tools	Didactic teaching / Lectures; Assessment / Feedback activities; Case-based learning; Role-play / Simulation; E-learning / Online platforms	Feedback surveys + exam results	Post	Faculty-led evaluation	Knowledge	Exam/Performance-based assessment	Level 1, Level 2b, Level 2c	Positive: High satisfaction; equal exam performance to pre-COVID; online teaching effective for mental health education.
OB04	Y	Role-play / Simulation	Simulation-based learning	Role-play / Simulation; Assessment / Feedback activities	Prospective cohort study (pre/post survey)	follow-up	Researcher-led evaluation	Knowledge	Mental Health Literacy Beliefs Questionnaire	Level 2b, Level 2c, Level 3	Positive: Simulation improved knowledge and sustained acceptance of treatment interventions; long-term retention observed.
OB05	Y	Role-play / Simulation; Reflective Exercises	Simulation-based learning	Role-play / Simulation; Assessment / Feedback activities; Mindfulness / Reflective activities	Cross-sectional survey (US & Turkey)	Post	Faculty-led evaluation	Self-Efficacy & Confidence	Simulation Effectiveness Tool – Modified (SET-M); Student Satisfaction & Self-Confidence in Learning Scale (NLN Instrument)	Level 1, Level 2c	Positive: Increased satisfaction and self-confidence; SP simulations effective globally.
OB06	Y	Real-world / Clinical Contact	Not reported	Clinical placements / Practical experience; Didactic teaching / Lectures	Longitudinal repeated-measures survey	Baseline, post, follow-up	Researcher-led evaluation	Attitudes & Stigma	Escala de Medición de Actitudes Hacia los Enfermos Mentales en Futuros Técnicos de Salud; Mental Illness Clinicians' Attitudes Scale; Reported and	Level 2a, Level 3	Positive: Clinical exposure improved attitudes & behaviors; stigma decreased especially in younger students.
OB07	Y	Role-play / Simulation	Not reported	Didactic teaching / Lectures; Workshops / Interactive sessions; Group discussions / Peer learning	Pre/post surveys (school + university students)	Baseline, post	Program facilitator assessment	Knowledge	Developed survey/questionnaire	Level 2a, Level 2b, Level 3	Positive: Improved knowledge & attitudes; reduced stigma; strong acceptability; sustained peer-led model.
OB08	Y	Role-play / Simulation	Video / Film materials	Didactic teaching / Lectures; Video / multimedia; Role-play / Simulation	Pre/post surveys + follow-up	post , follow-up	Faculty-led evaluation	Knowledge	Developed survey/questionnaire	Level 2b, Level 2c, Level 3	Positive: Significant gains in knowledge & practical skills; long-term retention noted by counseling feedback.
RCT01	Y	Role-play / Simulation	Online / e-learning platforms	Didactic teaching / Lectures; Video / multimedia; Assessment / Feedback activities; Case-based learning	Randomized crossover RCT (baseline, 10w, 24w follow-ups)	Baseline, follow-up	Unspecified / Not reported	Self-Efficacy & Confidence	Suicide Intervention Response Inventory (Version 2), Objective Structured Video Examination, Brief Resilience Scale, Perception of Academic	Level 2a, Level 2b, Level 2c	Positive: Improved knowledge & self-efficacy; sustained effects at 24 weeks.
RCT02	Y	Role-play / Simulation	Not reported	Case-based learning; Group discussions / Peer learning	Pre/post knowledge test + qualitative feedback	Baseline,post	Mixed evaluation (faculty & researcher)	Knowledge	Exam/Performance-based assessment	Level 2b, Level 2c	Positive: Cooperative PBL improved knowledge & teamwork; students valued collaboration.
RCT03	Y	Role-play / Simulation	Not reported	Psychoeducation / Communication training	Online pre/post/follow-up surveys	Baseline, post, follow-up	Student self-assessment,Researcher led evaluation	Knowledge	Mental Health First Aid Knowledge Test / Course Evaluation	Level 2a, Level 2b, Level 2c	Positive: Increased knowledge & confidence, reduced stigma; effects maintained at 2 months.
RCT05	Y	Role-play / Simulation	Online / e-learning platforms; Video / Film materials; Surveys / interactive tools	Didactic teaching / Lectures; Video / multimedia; E-learning / Online platforms; Workshops / Interactive sessions	Randomized pre/post survey	Baseline, follow-up	Student self-assessment	Knowledge	Mental Health First Aid Knowledge Test / Course Evaluation; Opening Minds Scale for Health Care Providers	Level 2a, Level 2b, Level 2c	Positive: Improved MHFA skills, reduced stigma, increased confidence & peer support.
RCT06	Y	Mindfulness / Self-reflection	Video / Film materials	Mindfulness / Reflective activities; Didactic teaching / Lectures; Group discussions / Peer learning	RCT pre/post self-report measures	Baseline, post	External evaluation	Mental Health & Wellbeing	Five Facet Mindfulness Questionnaire; General Health Questionnaire; Maslach Burnout Inventory–Student Survey; Perceived Medical School Stress	Level 2a, Level 2b, Level 2c, Level 3	Positive: Reduced distress & improved mindfulness; greater benefit for female students.
RCT07	Y	Real-world / Clinical Contact	Video / Film materials	Didactic teaching / Lectures; Patient / Service user involvement	Pre/post/follow-up MANOVA	Baseline, post, follow-up	Student self-assessment,Researcher led evaluation	Attitudes & Stigma	Opening Minds Scale for Health Care Providers; Opening Minds Scale for Health Care Providers (subscales)	Level 2a, Level 2b	Positive: Significant reduction in stigma & social distance across both formats.
QS01	Y	Real-world / Clinical Contact	Not reported	Psychoeducation / Communication training	Pre/post cohort survey	Baseline, post	Faculty-led evaluation	Self-Efficacy & Confidence	Attitudes Toward Psychiatry Scale-30, Developed survey/questionnaire	Level 2a, Level 2b, Level 2c	Positive: MHFA improved attitudes & confidence; better preparedness for practice.

QSI02	http://www.biomedcentral.com/1472-6920/11/51	Aliya Kassam, Nick Glozier, Morven Leese, Joanne Loughran, Graham Thornicroft |	2011	BMC Medical Education (2011)	United Kingdom	European Region (EUR)	University classroom	Single	Quasi-experimental	Medical students	Uniprofessional	Undergraduate	110 students completed both pre- and post-intervention assessments (out of 408 eligible). |	Cluster Sampling	Stigma Reduction Training Package	Multi-method Approach	Very short-term	Face-to-face	Interdisciplinary Team (Mixed roles)	Social and personal impacts of stigma and discrimination in areas of healthcare, employment, civil society, personal relationships, medication, media, and aggression. |	Evaluate mental illness stigma training for medical students.	Y	Y	Y	Y	N	Y	Y	Y	Role-play / Simulation	Not reported	Didactic teaching / Lectures; Patient / Service user involvement; Role-play / Simulation	Non-randomized controlled pre/post	Pre/post-program	External evaluation	Knowledge	Attitudes Toward Psychiatry Scale-30; Mental Health Knowledge Schedule	Level 2a, Level 2b, Level 3	Positive: Improved knowledge; no immediate attitude or behavior change; knowledge must be reinforced.
QSI03	https://doi-org.uaeu.idm.oclc.org/10.3109/00048674.2011.585454	Claire L. O'Reilly, J. Simon Bell, Patrick J. Kelly, Timothy F. Chen |	2011	Australian and New Zealand Journal of Psychiatry (2011)	Australia	Western Pacific Region (WPR)	University classroom	Single	Quasi-experimental	Pharmacy students	Uniprofessional	Undergraduate	MHFA Group:** n=60 (randomly selected from applicants).**Control Group:** n=252 (114 applicants not selected + 98 non-applicants).	Census sampling	Mental Health First Aid for Pharmacy Students	workshop/training	Very short-term	Face-to-face	Interdisciplinary Team (Mixed roles)	Symptoms, causes, and evidence-based treatments for depression, anxiety disorders, psychosis, and substance use disorder. Crisis situations (suicidal feelings, panic attacks, trauma, acute psychosis, overdose).	Examine MHFA's impact on pharmacy students' literacy and stigma.	Y	Y	Y	Y	Y	Y	Y	Y	Role-play / Simulation	Video / Film materials	Didactic teaching / Lectures; Case-based learning; Video / multimedia; Group discussions / Peer learning	Non-randomized pre/post	Baseline, post	Researcher-led evaluation	Knowledge	Mental Health First Aid Knowledge Test / Course Evaluation, Social Distance Scale	Level 2a, Level 2b, Level 2c	Positive: Increased recognition & confidence; reduced stigma; improved professional behavior.
QSI04	https://doi.org/10.1038/s41598-025-15633-8	Yasser M. Alotaibi, Latifah Alenezi, Fiddah M. Ahmad, Wadha Almutairi, Dhuha Alshammari, Manal Alenezi, Moudhi Alshatti	2025	Scientific Reports (2025)	Kuwait	Eastern Mediterranean Region (EMR)	University classroom	Single	Quasi-experimental	Medicine students, Dentistry students, Pharmacy students, Occupational Therapy students, Physical Therapy students, Radiologic Science	Multi-professional	Undergraduate	98 (Intervention: 56, Control: 42)	Convenience	Stress Management Educational Seminar	Multi-method Approach	Short-term	Face-to-face	Faculty / Instructor	Stress management, depression, anxiety, coping strategies, self-care, help-seeking, CBT, mindfulness	Evaluate culturally adapted stress management program for health science students.	Y	Y	Y	Y	Y	Y	Y	Y	Reflective Exercises	Not reported	Mindfulness / Reflective activities; Psychoeducation / Communication training	Quasi-experimental pre/post	Baseline, post, follow-up	External evaluation	Mental Health & Wellbeing	Depression, Anxiety and Stress Scale (21-item); Perceived Stress Scale	Level 2a, Level 2b, Level 2c, Level 3	Positive: Improved stress & depression; high satisfaction (82% useful, 89% would recommend).
QSI05	https://doi.org/10.1007/s42399-022-01225-x	Yasuhiro Kotera, Ann-Marie Edwards, Guilcan Garip, James Chircop, Muhammad Aledeh |	2022	SN Comprehensive Clinical Medicine (2022)	United Kingdom	European Region (EUR)	Online environment	Single	Quasi-experimental	Psychology students, Nursing students, Counselling students	Multi-professional	Undergraduate,Postgraduate	28 students completed all assessments (18 in mental health classes, 12 in non-mental health classes).	Convenience	Online Mental Health Class (MOOC-style)	Online / E-learning Modules	Extended/Continuous	Online/e-learning	Faculty / Instructor	Diagnosing mental disorders (DSM-5/ICD-11), social model of mental illness, stigma and shame, mental health practitioners' accounts and self-care. |	Evaluate online mental health classes for stigma reduction & well-being.	Y	Y	Y	Y	Y	Y	Y	Y	Not reported	Online / e-learning platforms	E-learning / Online platforms	Controlled before-after study	Pre, post, follow-up	Researcher-led evaluation	Attitudes & Stigma	Depression Stigma Scale; Depression, Anxiety and Stress Scale (21-item)	Level 2a, Level 2b	Positive: Stigma reduced post-semester; continuous education needed for lasting effect.
QSI06	https://doi.org/10.1186/s12909-021-02990-4	Marja Kurki, Sonja Gilbert, Kaisa Mishina, Lotta Lempinen, Terhi Luntamo, Susanna Hinkka-Yli-Salomäki, Atte Sinokki, Subina Upadhyaya, Yifeng Wei, Andre Sourander |	2021	BMC Medical Education (2021)	Finland	European Region (EUR)	University classroom	Single	Quasi-experimental	Nursing students, Medicine students, Dentistry students, Pharmacy students, Applied Medical Sciences students	Multi-professional	Undergraduate	158 students completed all stages (baseline, post-test, follow-up) |	Convenience	Digital MHL Program "Transitions"	Online / E-learning Modules	Very short-term (repeated)	Blended	Licensed Mental Health Clinician	Independent life skills, finances, accommodation, study skills, relationships,Mental health as a resource, stress management, crises, intoxicants., Mental disorders (depression, anxiety, eating disorders, etc.), help-seeking, treatment. |	Assess 'Transitions' digital MHL program for medical students' wellbeing	Y	Y	Y	Y	Y	Y	Y	Y	Mindfulness / Self-reflection	Online / e-learning platforms; Video / Film materials	Didactic teaching / Lectures; Video / multimedia; Mindfulness / Reflective activities; E-learning / Online platforms	One-group repeated measures	Pre, post, follow-up	Researcher-led evaluation	Knowledge	General Health Questionnaire; Mental Health Literacy Scale; Perceived Stress Scale	Level 2a, Level 2b, Level 2c	Positive: Improved knowledge & stress reduction; attitudes improved; stigma unchanged.
QSI07	https://doi.org/10.3390/ijerph19095269	Hsuan-Jung Lai, Yin-Ju Lien, Kai-Ren Chen, Yu-Kai Lin	2022	International Journal of Environmental Research and Public Health (2022)	Taiwan	Western Pacific Region (WPR)	University classroom	Two-site	Quasi-experimental	Public Health students	Uniprofessional	Undergraduate	48 participants (completed pre, post, and follow-up). |	Convenience	Mental Health Literacy (MHL) Curriculum	Integrated Curriculum Course	Long-term	Blended	Licensed Mental Health Clinician	Maintenance of positive mental health (self-concept, stress/emotion management). Recognition of mental illness (symptoms, epidemiology of depression, anxiety, schizophrenia, etc.).Reduction of mental illness stigma. Help-seeking efficacy and attitude (resources and skills). |	Examine effects of an 18-week MHL curriculum for public health students.	Y	Y	Y	Y	Y	Y	Y	Y	Real-world / Clinical Contact	Video / Film materials	Didactic teaching / Lectures; Video / multimedia; Group discussions / Peer learning	Pre/post/follow-up	Baseline, post,follow-up	Faculty-led evaluation	Attitudes & Stigma	MHL Scale for Healthcare Students	Level 2a, Level 2b	Positive: Increased MHL knowledge & reduced stigma; effects maintained at 6 weeks.
QSI08	https://doi.org/10.1186/s12909-022-03879-6	Rodríguez-Ferrer, J. M.; Manzano-León, A.; Fernández-Jiménez, C.; Aguilar-Parra, J. M.; Cangas, A. J.; Luque de la Rosa, A.	2022	BMC Medical Education (2022)	Spain	European Region (EUR)	University classroom	Two-site	Quasi-experimental	Nursing students	Uniprofessional	Undergraduate	253 students (Experimental group: n=138; Control group: n=115) |	Convenience	Digital Escape Room in Nursing Education	Simulation-based Education	Very short-term	Online/e-learning	Faculty / Instructor	Awareness and characteristics of Severe Mental Illness (SMI), reducing stigma and discriminatory attitudes (dangerousness, fear, coercion, avoidance). |	Evaluate gamified digital escape room for mental health stigma reduction.	Y	Y	Y	Y	Y	Y	Y	Y	Role-play / Simulation	Simulation-based learning; Online / e-learning platforms; Video conferencing tools	Role-play / Simulation; Workshops / Interactive sessions; Mindfulness / Reflective activities	Pre/post/follow-up	Baseline, post,follow-up	Mixed evaluation (faculty & researcher)	Attitudes & Stigma	Stigma Sensitization Scale	Level 2a, Level 2b, Level 3	Positive: Reduced stigma; long-term retention (6 months) confirmed; engaging learning approach.
QSI09	https://doi.org/10.1016/j.apnu.2025.01.005	Abeer Selim a,* , Christopher Newby b,1 , Anwar Almutairy c, Abdulrahman Aldossari d, Fahad Alkabba e, Saeed Arabi d, Mohamed Ali Zoromba f,g , Mohamed Hussein Ramadan Atta h,i , Nashwa Ibrahim	0	Archives of Psychiatric Nursing (2025)	Saudi Arabia	Eastern Mediterranean Region (EMR)	University classroom	Multi-site	Quasi-experimental	Nursing students, Medical students, Dentistry students, Pharmacy students, Applied Medical Sciences students	Multi-professional	Undergraduate	493 participants	Convenience	Digital Warning Signs (DWS) MHL Program	Simulation-based Education	Very short-term	Blended	Faculty / Instructor	11 warning signs of mental illness, early intervention, treatment options |	Evaluate digital-based mental health literacy intervention for early detection skills.	Y	Y	Y	Y	Y	Y	Y	Y	Role-play / Simulation	Video / Film materials; Surveys / interactive tools	Video / multimedia; Case-based learning; Group discussions / Peer learning	One-group pre/post	Baseline, post	Researcher-led evaluation	Knowledge	Developed survey/questionnaire	Level 2a, Level 2b	Positive: Knowledge & attitudes increased significantly; effective scalable program.
QSI10	https://doi.org/10.12688/f1000research.111002.2	Asumi Takahashi, Hirokazu Tachikawa, Ayumi Takayashiki, Takami Maeno, Yuki Shiratori, Asaki Matsuzaki, Tetsuaki Arai	2023	F1000Research (2023)	Japan	Western Pacific Region (WPR)	University classroom	Single	Quasi-experimental	Medical students	Uniprofessional	Undergraduate	136 attended. 121 completed pre/post questionnaires. 107 completed the 6-month follow-up. |	Convenience	CAMPUS Program (Crisis-Management, Anti-Stigma, MHL)	Multi-method Approach	Very short-term	Face-to-face	Licensed Mental Health Clinician	Stress, depression, suicidal thoughts, self-care for mental health issues, support resources, suicide risk assessment, listening skills, referral to support, combating self-stigma, misunderstandings about suicide. |	Evaluate suicide prevention & MHL program for university students.	Y	Y	Y	Y	Y	Y	Y	Y	Role-play / Simulation	Video / Film materials	Didactic teaching / Lectures; Video / multimedia; Role-play / Simulation; Group discussions / Peer learning	Pre/post/follow-up	Baseline, follow-up	External evaluation	Skills	Developed survey/questionnaire	Level 2a, Level 2b, Level 2c, Level 3	Positive: Reduced suicidal thoughts, improved self-efficacy & help-seeking intentions.
QSI11	https://doi-org.uaeu.idm.oclc.org/10.1080/08897077.2011.640151	| T. Bradley Tanner, Susan E. Wilhelm, Karen M. Rossie, Mary P. Metcalf |	2012	Substance Abuse (2012)	United States	Region of the Americas (AMR)	Online environment	Two-site	Quasi-experimental	Nursing students, Medical students	Multi-professional	not specified	Medical students: n = 10<• Nursing students: n = 60<• Primary care providers: n = 65 (evaluation in progress) |	Convenience	Web-Based SBIRT Skills Training	Online / E-learning Modules Case-based Education	Unspecified (not reported)	Online/e-learning	Self-guided / No human facilitator	| Program 1 (Students): Screening, assessment, diagnosis of alcohol abuse/dependence; brief intervention; treatment referral; communication and coordinated care.<Program 2 (Providers): Screening for hazardous substance use; brief intervention and other treatments; referral to treatment and follow-up care; SBIRT in practice. |	Evaluate a web-based SBIRT training program for health students.	Y	Y	Y	Y	Y	Y	Y	Y	Role-play / Simulation; Reflective Exercises	Simulation-based learning; Online / e-learning platforms; Video conferencing tools	Role-play / Simulation; Case-based learning; Assessment / Feedback activities; E-learning / Online platforms	Pre/post web-based evaluation	Baseline, post	External evaluation	Knowledge	Online standardized patient & SBIRT scales	Level 2a, 2b, 2c	Positive: Increased SBIRT knowledge, confidence, and clinical competence.
QSI12	https://doi.org/10.1186/s12909-023-05019-0	Benedikt Till, Angelika Hofhansl, Thomas Niederkrotenthaler |	2024	BMC Medical Education (2024)	Austria	European Region (EUR)	University classroom	Single	Quasi-experimental	Medical students	Uniprofessional	Undergraduate	137 participants (from 3,975 invited) |	Convenience	Coping with Stress Seminar (Onsite/Online)	Seminar	Very short-term	Blended	Faculty / Instructor	Stress, burnout, coping strategies, suicidal ideation, help resources |	Examine effects of 'Coping with Stress' seminar for first-year medical students.	Y	Y	Y	Y	Y	Y	Y	Y	Reflective Exercises	Online / e-learning platforms	Didactic teaching / Lectures; Group discussions / Peer learning; Mindfulness / Reflective activities; Assessment / Feedback activities	Pre/post comparison among cohorts	Baseline, post	Mixed evaluation (faculty & researcher)	Knowledge	Burnout Assessment Tool, Satisfaction With Life Scale, Stress and Coping Inventory (Physical and Psychological Stress Subscale), Developed	Level 2a, 2b, 3	Positive: Onsite seminar reduced stress & burnout; improved help-seeking knowledge.
QSI13	https://doi.org/10.4088/PCC.17m02210	Andreea L. Seritan, Donald M. Hilty, Ana-Maria Iosif, Peter J. Ureste, Tammy L. Duong	2017	Primary Care Companion for CNS Disorders (2017)	United States	Region of the Americas (AMR)	Hospital	Single	Quasi-experimental	Family Medicine Residents	Uniprofessional	Postgraduate	24 eligible; 15 completed both surveys	Convenience	Resident-Led Psychiatry Education (6-hour)	Lecture-based Education	Very short-term	Face-to-face	Licensed Mental Health Clinician	Psychiatric emergencies, capacity assessment, delirium, bipolar disorder, psychosis, depression, anxiety	Assess a resident-led mental health intervention for family medicine residents.	Y	Y	Y	Y	Y	Y	Y	Y	Role-play / Simulation	Not reported	Didactic teaching / Lectures; Case-based learning; Group discussions / Peer learning	Pre/post surveys	Baseline, post	Peer-led evaluation, Faculty-led evaluation	Knowledge	Developed survey/questionnaire	Level 2a, 2b, 2c	Positive: Increased comfort managing psychiatric comorbidities; high satisfaction.
QSI14	https://doi.org/10.3390/nursrep14040216	Olga Valentim, Tânia Correia, Lídia Moutinho, Paulo Seabra, Ana Querido, Carlos Laranjeira	2024	Nursing Reports (2024)	Portugal	European Region (EUR)	University classroom	Single	Quasi-experimental	Nursing students	Uniprofessional	Undergraduate	37 students	Convenience	"This Is Me" Psychoeducational Program	workshop/training	Very short-term	Face-to-face	Faculty / Instructor	Stigma, discrimination, mental health rights, empathy	Evaluate 'This Is Me' anti-stigma program for nursing students.	Y	Y	Y	Y	Y	Y	Y	Y	Real-world / Clinical Contact; Reflective Exercises	Not reported	Didactic teaching / Lectures; Group discussions / Peer learning; Role-play / Simulation; Patient / Service user involvement	Pre/post intervention (questionnaires)	Baseline, post	Researcher-led evaluation	Skills	Attribution Questionnaire – 27 Item; Interpersonal Attraction Scale; Jefferson Scale of Physician Empathy; Mental Health Knowledge Schedule;	Level 2a, 2b, 2c	Positive: Increased empathy, reduced stigma & anxiety; improved perspective taking.
QSI15	https://doi.org/10.1186/s12909-020-02438-1	Qinghua Wang and Tianjiao Du	2020	BMC Medical Education (2020)	China	Western Pacific Region (WPR)	University classroom	Single	Quasi-experimental	Medical students	Uniprofessional	Undergraduate	374 (188 experiment, 186 control)	Mixed / Quota sampling	College Student Mental Health Education Course (CSMHEC)	Integrated Curriculum Course	Medium-term	Face-to-face	Licensed Mental Health Clinician	Stress, anxiety, depression, burnout, emotion regulation, self-awareness, personality development	Evaluate effectiveness of 8-week mental health education in medical curriculum.	Y	Y	Y	Y	Y	Y	Y	Y	Reflective Exercises	Surveys / interactive tools	Didactic teaching / Lectures; Mindfulness / Reflective activities	Pre/post intervention + thematic analysis	Baseline, post	Faculty-led evaluation	Mental Health & Wellbeing	Compassionate Care Scale; Depression, Anxiety and Stress Scale (21-item); Satisfaction With Life Scale	Level 2a, 2b, 3	Positive: Reduced distress & burnout; increased life satisfaction & course satisfaction.
QSI16	https://doi-org.uaeu.idm.oclc.org/10.1097/01.nep.0000000000000796	Sawsan Abuhammad and Shaher Hamaideh	2022	Nursing Education Perspectives (2021)	Jordan	Eastern Mediterranean Region (EMR)	University classroom	Single	Quasi-experimental	Nursing students	Uniprofessional	Undergraduate	205 students (150 female, 55 male) |	Convenience	Comprehensive Mental Health Course	Integrated Curriculum Course	Long-term	Face-to-face	Faculty / Instructor	0	Compare nursing students' attitudes toward help-seeking pre/post mental health course	Y	Y	Y	Y	Y	Y	Y	Y	Real-world / Clinical Contact	Not reported	Didactic teaching / Lectures; Clinical placements / Practical experience	Pre/post questionnaire design	Baseline,follow-up	Faculty-led evaluation	Attitudes & Stigma	Attitudes Toward Seeking Professional Help Scale	Level 2a, 2b	Positive: Significant improvement in help-seeking attitudes & awareness.
QSI17	https://doi.org/10.1371/journal.pone.0319431	Felipe Agudelo-Hernández, Helena Vélez-Botero, Marcela	2025	PLOS ONE (2025)	Colombia	Region of the Americas (AMR)	University classroom	Single	Quasi-experimental	Medical students	Uniprofessional	Undergraduate	194 students	Convenience	WHO QualityRights Training	workshop/training	Medium-term	Blended	Interdisciplinary Team (Mixed roles)	Human rights, mental health, disability, recovery, coercion, community inclusion |	Evaluate human rights & stigma education for medical students.	Y	Y	Y	Y	Y	Y	Y	Y	Real-world / Clinical Contact	Online / e-learning platforms	Human rights / Legal / Ethics content	Pre/post evaluation	Baseline, post	External evaluation	Attitudes & Stigma	Community Attitudes toward the Mentally Ill; Health-Related Engagement Scales; Mental Illness Clinicians' Attitudes Scale	Level 2a, 2b, 3	Positive: Improved rights understanding, reduced stigma & authoritarianism.
QSI18	https://doi-org.uaeu.idm.oclc.org/10.1186/s40359-015-0069-0	Kathy S Bond, Anthony F Jorm, Betty A Kitchener, Nicola J Reavley |	2015	BMC Psychology (2015)	Australia	Western Pacific Region (WPR)	University classroom	Single	Quasi-experimental	Nursing students, Medical students	Multi-professional	Undergraduate,Postgraduate	434 (292 nursing, 142 medical) |	Mixed / Quota sampling	Tailored MHFA Training (Face-to-Face & Online)	workshop/training	Very short-term	Blended	External Trainer / Consultant	Recognising mental health problems (e.g., depression, eating disorders), crisis assessment, providing initial help, listening non-judgmentally, giving support and information, encouraging professional help and other supports. |	Assess MHFA training's effectiveness in medical & nursing students.	Y	Y	Y	Y	Y	Y	Y	Y	Role-play / Simulation	Online / e-learning platforms; Video / Film materials	Didactic teaching / Lectures; Video / multimedia; Workshops / Interactive sessions	Pre/post surveys	Baseline, post	Researcher-led evaluation	Self-Efficacy & Confidence	Mental Health First Aid Knowledge Test / Course Evaluation	Level 2a, 2b, 2c	Positive: Improved MHFA literacy, reduced stigma; both online & onsite effective.
QSI19	https://doi-org.uaeu.idm.oclc.org/10.1111/j.1365-2850.2009.01421.x	F. Brown	2009	Journal of Psychiatric and Mental Health Nursing (2009)	United States	Region of the Americas (AMR)	Community	Multi-site	Quasi-experimental	Nursing students	Uniprofessional	Undergraduate	Students:** 55 (preliminary) + 35 (study) = 90 total.<>**Community Participants:** 38 (with matched pre/post data for analysis). |	Convenience	Faith-Based Mental Health Education Program	workshop/training	Very short-term	Face-to-face	Peer Facilitator (Student)	General information about the etiology, signs, symptoms, treatment, and supportive resources for a variety of mental disorders and substance abuse problems. Tailored topics included depression and dementia	Evaluate service-learning in faith-based community education for nursing students	Y	Y	Y	Y	Y	Y	Y	Y	Real-world / Clinical Contact	Video / Film materials	Didactic teaching / Lectures; Video / multimedia; Assessment / Feedback activities; Workshops / Interactive sessions	Pre/post reflection & observation	post	Mixed evaluation (faculty & researcher)	Attitudes & Stigma	Observation/Reflection tool	Level 2a, 2b, 3	Positive: Increased knowledge & skills; reduced stigma; enhanced service-learning benefits.
QSI20	https://doi-org.uaeu.idm.oclc.org/10.1097/01.nep.0000000000001303	Vicki Gamez and Thomas Gunning |	2024	Nursing Education Perspectives (2024)	United States	Region of the Americas (AMR)	University classroom	Single	Quasi-experimental	Nursing students	Uniprofessional	Undergraduate	376 students (205 in pre-intervention group, 171 in post-intervention group). |	Convenience	Integrated Mental Health Curriculum	Integrated Curriculum Course	Extended/Continuous	Face-to-face	Faculty / Instructor	Recognizing, managing, and preventing mental illness; awareness of bias and stigma; therapeutic care. |	Examine integration of mental health across 2-year nursing curriculum.	Y	Y	Y	Y	Y	Y	Y	Y	Role-play / Simulation; Real-world / Clinical Contact	Not reported	Role-play / Simulation; Clinical placements / Practical experience	Pre/post evaluation	post	Faculty-led evaluation	Knowledge	Developed survey/questionnaire, Observation/Reflection tool	Level 2a, 2b, 2c, 3	Positive: Improved MHL, confidence, empathy; greater readiness for mental health practice.
QSI21	https://doi.org/10.1016/j.nepr.2021.103009	Libin Gu, Wenjuan Jiao, Haozhi Xia, Mingming Yu |	2021	Nurse Education in Practice (2021)	China	Western Pacific Region (WPR)	University classroom	Single	Quasi-experimental	Nursing students	Uniprofessional	Undergraduate	343 students (completed the study). |	Convenience	Psychiatric-Mental Health Education with Role-Play and Real-World Contact	Integrated Curriculum Course	Medium-term	Face-to-face	Interdisciplinary Team (Mixed roles)	Etiology, symptoms, psychopharmacology, nursing interventions for common disorders (substance abuse, schizophrenia, bipolar), legal/ethical issues, stigmatization, family/society burden, mental health promotion. |	Evaluate impact of psychiatric-mental health education integrating role-play and real-world contact	Y	Y	Y	Y	Y	Y	Y	Y	Role-play / Simulation; Real-world / Clinical Contact	Surveys / interactive tools	Didactic teaching / Lectures; Role-play / Simulation; Clinical placements / Practical experience; Mindfulness / Reflective activities	Single-group pre/post design	Baseline, post	Faculty-led evaluation	Attitudes & Stigma	SPMI (Stigma Towards People with Mental Illness Scale)	Level 2a, Level 2b, Level 3	Positive: Stigma reduced; willingness to care increased; effective blended role-play and contact model.
QSI22	http://dx.doi.org/10.1016/j.nedt.2015.09.007	Agnes Higgins, Margaret Carroll, Danika Sharek	2016	Nurse Education Today (2016)	Ireland	European Region (EUR)	University classroom	Single	Quasi-experimental	Midwives Students	Uniprofessional	Undergraduate	Pre-survey: n=28; Post-survey: n=26; Matched pairs for analysis: n=25 |	Convenience	Perinatal Mental Health Module (30 hours)	workshop/training	Very short-term	Face-to-face	Faculty / Instructor	Mental health problems (depression, anxiety, eating disorders, psychosis, bipolar disorder>Impact of mental health problems on mother and baby<>Risk factors<>Screening tools (e.g., Edinburgh Depression Scale)<>Use of psychotropic medication in pregnancy/breastfeeding>Maternal morbidity & suicide<>Support services<>Mental Health Act>Communication skills for sensitive topics	Assess perinatal mental health module's impact on midwifery students' knowledge, skills, and attitudes.	Y	Y	Y	Y	Y	Y	Y	Y	Reflective Exercises	Not reported	Psychoeducation / Communication training; Group discussions / Peer learning	Pre/post repeated-measures survey	Baseline, post	Faculty-led evaluation	Knowledge	Developed survey/questionnaire	Level 2a, Level 2b, Level 2c	Positive: Improved knowledge, skills, and attitudes; students reported increased confidence discussing PMH.
QSI23	0260-6917/© 2015 Elsevier Ltd. All rights reserved.	Luís Manuel de Jesus Loureiro, Lúcia Manuela Brandão Valente da Costa |	2019	Revista de Enfermagem Referência (2019)	Portugal	European Region (EUR)	University classroom	Single	Quasi-experimental	Nursing students	Uniprofessional	Undergraduate	| n = 46 |	Random sampling	Mental Health First Aid (Portuguese version)	workshop/training	Very short-term	Face-to-face	Faculty / Instructor	The program covers multiple disorders, but this study focused its evaluation specifically on depression	Evaluate MHFA program's effect on nursing students' mental health literacy.	Y	Y	Y	Y	Y	Y	Y	Y	Role-play / Simulation	Not reported	Psychoeducation / Communication training	Pre/post intervention	Baseline, post	External evaluation	Knowledge	QuALiSMental Questionnaire	Level 2a, Level 2b	Positive: Improved MHL and confidence; enhanced ability to recognize depression and provide support.
QSI24	https://doi.org/10.1007/s00737-023-01386-1	Lindsay Marty, Olivia Myrick, Allison Perelman, Amalia Kotlyar, Jessica Vernon |	2024	Archives of Women's Mental Health (2024)	United States	Region of the Americas (AMR)	University classroom	Single	Quasi-experimental	Maternal Residents	Uniprofessional	Postgraduate	Pre-intervention: n=20 (50% response rate); Post-intervention: n=9 (23% response rate) |	Census sampling	10-hour Perinatal Mental Health Lecture Series	Online / E-learning Modules	Medium-term	Online/e-learning	Interdisciplinary Team (Mixed roles)	| Fundamental principles of reproductive psychiatry, pharmacology, diagnostic tools, collaborative care models, care for BIPOC patients, trauma-informed care, disordered eating, substance use disorders in pregnancy. |	Assess pilot lecture series on perinatal mental health in OBGYN residency training.	Y	Y	Y	Y	Y	Y	Y	Y	Not reported	Video conferencing tools	Didactic teaching / Lectures; Group discussions / Peer learning	Cross-sectional pre/post Qualtrics survey	Baseline, post	Faculty-led evaluation	Knowledge	Developed survey/questionnaire	Level 2a, Level 2b	Positive: Increased comfort diagnosing PMADs; improved knowledge of screening tools; treatment confidence limited.
QSI25	https://doi-org.uaeu.idm.oclc.org/10.1080/08897077.2011.640103	Jason M. Satterfield, Patricia O'Sullivan, Derek D. Satre, Janice Y. Tsoh, Steven L. Batki, Kathy Julian, Elinore F. McCance-Katz, Maria Wamsley |	2012	Substance Abuse (2012)	United States	Region of the Americas (AMR)	University classroom; Clinical rotation	Single	Quasi-experimental	Internal Medicine Residents	Uniprofessional	Postgraduate	19 residents participated; 11 completed both pre and post assessments |	Convenience	10-hour SBIRT Curriculum (Screening, Brief Intervention, and Referral to Treatment)	workshop/training	Very short-term	Face-to-face	Licensed Mental Health Clinician	| Substance use screening, brief intervention, referral to treatment |	Evaluate SBIRT curriculum's effect on residents' substance use intervention skills	Y	Y	Y	Y	Y	Y	Y	Y	Role-play / Simulation; Reflective Exercises	Video / Film materials	Case-based learning; Psychoeducation / Communication training	Pre/post clinical skill evaluation	Baseline, follow-up	External evaluation	Knowledge	SBIRT Checklists	Level 2a, Level 2b, Level 2c, Level 3	Positive: Significant gains in SBIRT knowledge & skills; improved clinical communication & follow-up documentation.
QSI26	https://doi.org/10.5812/ijpbs.9672.	Saeed Vaghee, Azam Salarhaji, Masoud Kashani Lotfabadi, Nastaran Vaghei	2017	Iranian Journal of Psychiatry and Behavioral Sciences (2017)	Iran	Eastern Mediterranean Region (EMR)	Hospital	Single	Quasi-experimental	Nursing students	Uniprofessional	Postgraduate	111 students	Mixed / Quota sampling	Contact-Based & ACT Mental Health Training	workshop/training	Very short-term	Face-to-face	Licensed Mental Health Clinician	Stigma, recovery, social responsibility, disclosure	Compare contact-based education vs Acceptance and Commitment Training (ACT) to reduce stigma	Y	Y	Y	Y	Y	Y	Y	Y	Real-world / Clinical Contact; Reflective Exercises	Video / Film materials	Mindfulness / Reflective activities	Pre/post/follow-up	Baseline, follow-up	Faculty-led evaluation	Attitudes & Stigma	Opening Minds Scale for Healthcare Providers	Level 2a, Level 2b, Level 3	Positive: Both interventions reduced stigma; contact-based training improved recovery & social responsibility attitudes.
