# Supplementary material for: Mental health literacy training interventions for future health professionals: An evidence and gap mapping scoping review
Source: PLoS One. 2026 Oct 27;21(10):e0360403. doi: 10.1371/journal.pone.0360403 (PMC13712762; doi:10.1371/journal.pone.0360403)
Supplement: Supplementary file 2 [file pone.0360403.s001.docx]

**Title:** **Mental Health Literacy Training Interventions for Future Health Professionals: An Evidence and Gap Mapping Scoping Review**

| **Content list** | **Page No.** |
| --- | --- |
| **Supplementary 1A.** Search Strategies | 2-6 |
| **Supplementary 1B.** Standardized Coding Framework and Operational Definitions for Key Variables | 7-15 |
| **Supplementary 1C.** Included Studies | 16-21 |
| **Supplementary 1D.** Study design and outcome characteristics by WHO region | 22-23 |
| **Supplementary 1E.** Sample size distribution by study design | 24 |
| **Supplementary 1F.** Temporal trends in the adoption of experiential components and educational technology | 25 |
| **Supplementary 1G.** Theoretical frameworks and models applied in mental health literacy educational interventions for health professions students, categorized by framework type | 26 |
| **Supplementary 1H.** Topic Coverage Across Included Studies | 27 |
| **Supplementary 1I.** List of Assessment Scales and Instruments used in mental health literacy educational interventions for health professions students, categorized by scale category | 26-30 |
| **Supplementary 1J.** Evaluation Timing and Follow-up by Study Design | 31 |
| **Supplementary 1K.** Summary of Assessment Approaches | 31 |

**Supplementary 1A Search Strategies**

Population:

"health profession student*" OR "health student*" OR "medical student*" OR "nursing student*" OR "allied health" OR "health care student*" OR "healthcare student*" OR "health-care student*" OR "health professional education" OR “Undergraduate medical” OR “Undergraduate health” OR “Postgraduate medical” OR “Postgraduate health” OR “MBBS” OR “residents” OR “interns” OR “trainees”

Intervention/Exposure:

"mental health literacy" OR "mental health education*" OR "mental health training*" OR "psychoeducat*" OR "mental health aware*" OR "mental health promot*" OR "psychological literac*" OR "psychological aware*" OR "psychological educat*" OR "psychological training*" OR “Psychotherapy educat*”

Outcome:

"knowledge*" OR "attitude*" OR "skill*" OR "competenc*" OR "confidence*" OR "effecti*" OR "efficac*" OR "impact*" OR "evaluat*" OR "proficien*" OR "performanc*" OR "behavior*" OR "learning outcom*" OR "self-effic*"

Refining and search filtering:

Types of sources: Quantitative, qualitative, and mixed-method primary research studies.

Language: English

Date: 1997 to 2025

Excluding Animal studies

Pubmed:

Hits: 2,419 by 14/05/2025

Search: ((("Psychological Well-Being"[Mesh] OR "Psychotherapy/education"[Mesh] OR "Psychotherapy/methods"[Mesh] OR "mental health literacy"[Title/Abstract] OR "mental health education*"[Title/Abstract] OR "mental health training*"[Title/Abstract] OR "psychoeducat*"[Title/Abstract] OR "mental health aware*"[Title/Abstract] OR "mental health promot*"[Title/Abstract] OR "psychological literac*"[Title/Abstract] OR "psychological aware*"[Title/Abstract] OR "psychological educat*"[Title/Abstract] OR "psychological training*"[Title/Abstract] OR "Psychotherapy educat*"[Title/Abstract]) AND ("Knowledge"[Mesh] OR "Attitude"[Mesh] OR "Mental Competency"[Mesh] OR "Self Concept"[Mesh] OR "Program Evaluation"[Mesh] OR "Self-Evaluation Programs"[Mesh] OR "Nursing Evaluation Research"[Mesh] OR "Behavior"[Mesh] OR "knowledge*"[Title/Abstract] OR "attitude*"[Title/Abstract] OR "skill*"[Title/Abstract] OR "competenc*"[Title/Abstract] OR "confidence*"[Title/Abstract] OR "effecti*"[Title/Abstract] OR "efficac*"[Title/Abstract] OR "impact*"[Title/Abstract] OR "evaluat*"[Title/Abstract] OR "proficien*"[Title/Abstract] OR "performanc*"[Title/Abstract] OR "behavior*"[Title/Abstract] OR "learning outcom*"[Title/Abstract] OR "self-effic*"[Title/Abstract])) AND ("Students, Health Occupations"[Mesh] OR "Allied Health Occupations"[Mesh] OR "Health Education"[Mesh] OR "Education, Nursing, Graduate"[Mesh] OR "Education, Medical, Graduate"[Mesh] OR "Education, Medical"[Mesh] OR "Education, Nursing"[Mesh] OR "Education, Public Health Professional"[Mesh] OR "Internship, Nonmedical"[Mesh] OR "Interprofessional Education"[Mesh] OR "Mentoring"[Mesh] OR"health profession student*"[Title/Abstract] OR "health student*"[Title/Abstract] OR "medical student*"[Title/Abstract] OR "nursing student*"[Title/Abstract] OR "allied health"[Title/Abstract] OR "health care student*"[Title/Abstract] OR "healthcare student*"[Title/Abstract] OR "health-care student*"[Title/Abstract] OR "health professional education"[Title/Abstract] OR "Undergraduate medical"[Title/Abstract] OR "Undergraduate health"[Title/Abstract] OR "Postgraduate medical"[Title/Abstract] OR "Postgraduate health"[Title/Abstract] OR "MBBS"[Title/Abstract] OR "residents"[Title/Abstract] OR "interns"[Title/Abstract] OR "trainees"[Title/Abstract])) Filters: Case Reports, Classical Article, Clinical Trial, Clinical Trial, Phase I, Clinical Trial, Phase II, Clinical Trial, Phase III, Clinical Trial, Phase IV, Collected Work, Controlled Clinical Trial, Corrected and Republished Article, Evaluation Study, Introductory Journal Article, Multicenter Study, Observational Study, Overall, Randomized Controlled Trial, Technical Report, Twin Study, Validation Study, English, Humans, Exclude preprints, from 1997 - 2025

Scopus

( TITLE-ABS-KEY ( "health profession student*" OR "health student*" OR "medical student*" OR "nursing student*" OR "allied health" OR "health care student*" OR "healthcare student*" OR "health-care student*" OR "health professional education" OR "Undergraduate medical" OR "Undergraduate health" OR "Postgraduate medical" OR "Postgraduate health" OR "MBBS" OR "residents" OR "interns" OR "trainees" ) AND TITLE-ABS-KEY ( "mental health literacy" OR "mental health education*" OR "mental health training*" OR "psychoeducat*" OR "mental health aware*" OR "mental health promot*" OR "psychological literac*" OR "psychological aware*" OR "psychological educat*" OR "psychological training*" OR "Psychotherapy educat*" ) AND TITLE-ABS-KEY ( "knowledge*" OR "attitude*" OR "skill*" OR "competenc*" OR "confidence*" OR "effecti*" OR "efficac*" OR "impact*" OR "evaluat*" OR "proficien*" OR "performanc*" OR "behavior*" OR "learning outcom*" OR "self-effic*" ) ) AND PUBYEAR > 1996 AND PUBYEAR < 2026 AND ( LIMIT-TO ( LANGUAGE , "English" ) ) AND ( LIMIT-TO ( DOCTYPE , "ar" ) ) AND ( LIMIT-TO ( SRCTYPE , "j" ) )

Hits: 651 by 14/05/2025

WOS

"health profession student*" OR "health student*" OR "medical student*" OR "nursing student*" OR "allied health" OR "health care student*" OR "healthcare student*" OR "health-care student*" OR "health professional education" OR “Undergraduate medical” OR “Undergraduate health” OR “Postgraduate medical” OR “Postgraduate health” OR “MBBS” OR “residents” OR “interns” OR “trainees” (Topic) and "mental health literacy" OR "mental health education*" OR "mental health training*" OR "psychoeducat*" OR "mental health aware*" OR "mental health promot*" OR "psychological literac*" OR "psychological aware*" OR "psychological educat*" OR "psychological training*" OR “Psychotherapy educat*” (Topic) and "knowledge*" OR "attitude*" OR "skill*" OR "competenc*" OR "confidence*"OR "effecti*" OR "efficac*" OR "impact*" OR "evaluat*" OR "proficien*" OR "performanc*" OR "behavior*" OR "learning outcom*" OR "self-effic*" (Topic) and 1999 or 1998 or 2000 or 2001 or 2002 or 2003 or 2004 or 2005 or 2006 or 2007 or 2008 or 2009 or 2010 or 2011 or 2012 or 2013 or 2014 or 2025 or 2024 or 2023 or 2022 or 2021 or 2020 or 2019 or 2018 or 2017 or 2016 or 2015 (Publication Years) and English (Languages) and Article (Document Types)

Hits: 50 by 14/05/2025

Embase

('health profession student*':ti,ab,kw OR 'health student*':ti,ab,kw OR 'medical student*':ti,ab,kw OR 'nursing student*':ti,ab,kw OR 'allied health':ti,ab,kw OR 'health care student*':ti,ab,kw OR 'healthcare student*':ti,ab,kw OR 'health-care student*':ti,ab,kw OR 'health professional education':ti,ab,kw OR 'undergraduate medical':ti,ab,kw OR 'undergraduate health':ti,ab,kw OR 'postgraduate medical':ti,ab,kw OR 'postgraduate health':ti,ab,kw OR 'mbbs':ti,ab,kw OR 'residents':ti,ab,kw OR 'interns':ti,ab,kw OR 'trainees':ti,ab,kw) AND ('mental health literacy':ti,ab,kw OR 'mental health education*':ti,ab,kw OR 'mental health training*':ti,ab,kw OR 'psychoeducat*':ti,ab,kw OR 'mental health aware*':ti,ab,kw OR 'mental health promot*':ti,ab,kw OR 'psychological literac*':ti,ab,kw OR 'psychological aware*':ti,ab,kw OR 'psychological educat*':ti,ab,kw OR 'psychological training*':ti,ab,kw OR 'psychotherapy educat*':ti,ab,kw) AND ('knowledge*':ti,ab,kw OR 'attitude*':ti,ab,kw OR 'skill*':ti,ab,kw OR 'competenc*':ti,ab,kw OR 'confidence*':ti,ab,kw OR 'effecti*':ti,ab,kw OR 'efficac*':ti,ab,kw OR 'impact*':ti,ab,kw OR 'evaluat*':ti,ab,kw OR 'proficien*':ti,ab,kw OR 'performanc*':ti,ab,kw OR 'behavior*':ti,ab,kw OR 'learning outcom*':ti,ab,kw OR 'self-effic*':ti,ab,kw) #1 AND (1997:py OR 1998:py OR 1999:py OR 2000:py OR 2001:py OR 2002:py OR 2003:py OR 2004:py OR 2005:py OR 2006:py OR 2007:py OR 2008:py OR 2009:py OR 2010:py OR 2011:py OR 2012:py OR 2013:py OR 2014:py OR 2015:py OR 2016:py OR 2017:py OR 2018:py OR 2019:py OR 2020:py OR 2021:py OR 2022:py OR 2023:py OR 2024:py OR 2025:py) AND ([adolescent]/lim OR [adult]/lim OR [middle aged]/lim OR [young adult]/lim) AND 'article'/it

Hits: 203 by 14/05/2025

Ebscohost (ERIC, CINAHL Ultimate, APA PsycInfo)

SU ("health profession student*" OR "health student*" OR "medical student*" OR "nursing student*" OR "allied health" OR "health care student*" OR "healthcare student*" OR "health-care student*" OR "health professional education" OR Undergraduate medical OR Undergraduate health OR Postgraduate medical OR Postgraduate health OR MBBS OR residents OR interns OR trainees) AND SU ("mental health literacy" OR "mental health education*" OR "mental health training*" OR "psychoeducat*" OR "mental health aware*" OR "mental health promot*" OR "psychological literac*" OR "psychological aware*" OR "psychological educat*" OR "psychological training*" OR Psychotherapy educat*) AND SU ("knowledge*" OR "attitude*" OR "skill*" OR "competenc*" OR "confidence*" OR "effecti*" OR "efficac*" OR "impact*" OR "evaluat*" OR "proficien*" OR "performanc*" OR "behavior*" OR "learning outcom*" OR "self-effic*")

Hits: 134 by 14/05/2025

Psycarticles (Proquest)

subject("health profession student*" OR ("health students") OR ("medical student" OR "medical students") OR ("nursing student" OR "nursing students") OR "allied health" OR "health care student*" OR ("healthcare students") OR "health-care student*" OR "health professional education" OR "Undergraduate medical" OR "Undergraduate health" OR "Postgraduate medical" OR "Postgraduate health" OR "MBBS" OR "residents" OR "interns" OR "trainees") AND subject("mental health literacy" OR "mental health education*" OR "mental health training*" OR "psychoeducat*" OR "mental health aware*" OR "mental health promot*" OR "psychological literac*" OR "psychological aware*" OR "psychological educat*" OR ("psychological training") OR "Psychotherapy educat*") AND subject("knowledge*" OR "attitude*" OR "skill*" OR "competenc*" OR "confidence*" OR "effecti*" OR "efficac*" OR "impact*" OR "evaluat*" OR "proficien*" OR "performanc*" OR "behavior*" OR ("learning outcome" OR "learning outcomes") OR "self-effic*")

Hits: 4 by 14/05/2025

**Supplementary 1 B.** Standardized Coding Framework and Operational Definitions for Key Variables

**Note.** This table summarizes the standardized coding framework used to classify and analyze studies included in the systematic mapping review. Each coding category represents a distinct analytic variable (e.g., study design, intervention type, facilitator category, outcome domain) applied during data extraction and evidence mapping. Definitions were adapted from established evidence-and-gap-mapping methodologies (Bragge et al., 2011; Snilstveit et al., 2016) and refined inductively based on the characteristics of included studies.

| **Code Dimension** | **Coding Category** | **Operational Definition** |
| --- | --- | --- |
| **Study Design** | Randomized Controlled Trial (RCT) | Participants randomly allocated to intervention and control groups to test causal effects. |
|  | Quasi-experimental | Pre/post or comparison-group design without full randomization. |
|  | Mixed-methods | Combines quantitative and qualitative data collection and analysis. |
|  | Cross-sectional | Descriptive or correlational design using data collected at a single time point. |
|  | Qualitative | Exploratory, interpretive design using interviews, focus groups, or thematic analysis. |
|  | Cohort / Longitudinal | Follows participants over time to examine change or lasting impact. |
|  | Other / Not reported | Design unclear or not specified by authors. |
| **Sampling Method** | Random / Probability Sampling | Participants selected using randomization (simple, stratified, or cluster). |
|  | Convenience Sampling | Participants recruited based on accessibility or availability. |
|  | Purposive Sampling | Participants chosen intentionally for specific characteristics or expertise. |
|  | Census / Whole Cohort | Entire class, year level, or institution included. |
|  | Snowball / Chain Referral | Participants recruited through peer referral. |
|  | Not reported / Unclear | Sampling strategy not stated or insufficiently described. |
| **Type of Intervention** | **Lecture-based** | Traditional didactic delivery, often in classroom format, emphasizing instructor-led presentation of content with limited interaction. |
|  | **Workshop/Training** | Structured, skill-oriented sessions emphasizing participation, discussion, and practice, typically shorter-term. |
|  | **Simulation-based** | Learning through simulated or role-played scenarios (e.g., standardized patients, virtual simulations) to develop applied skills and confidence. |
|  | **Online / E-learning Modules** | Digitally delivered, asynchronous or synchronous content using online platforms, often including quizzes, videos, or discussion forums. |
|  | **Case-based Education** | Learner engagement through real or hypothetical clinical cases to apply theoretical knowledge to problem-solving. |
|  | **Contact-based Education** | Direct interaction with individuals with lived experience of mental illness (“Experts by Experience”) to foster empathy and reduce stigma. |
|  | **Problem-based Education** | Student-centered learning using open-ended mental health problems that encourage research, teamwork, and critical reasoning. |
|  | **Team-based Education** | Collaborative small-group approach emphasizing teamwork, discussion, and shared problem-solving. |
|  | **Arts-based Education** | Integration of creative media (e.g., film, theater, visual arts) to explore mental health themes and emotional engagement. |
|  | **Course / Multi-method Approach** | Comprehensive curricula combining two or more of the above modalities (e.g., lectures + workshops + e-learning). |
| **Mode of Delivery** | **Face-to-face** | Delivered in person, typically within classrooms, workshops, or simulation labs. |
|  | **Online / E-learning** | Entirely digital format (synchronous or asynchronous) using web platforms or apps. |
|  | **Blended** | Combination of face-to-face and online delivery. |
|  | **Simulation-based** | Delivered through live or virtual simulation environments. |
|  | **Experiential** | Learning through direct, reflective experience (e.g., clinical placements, role-play, mindfulness exercises). |
|  | **Not specified** | Delivery mode unclear or not reported in the study. |
| **Intervention Duration** | **Very short-term (≤1 week)** | Single session or intervention lasting one week or less. |
|  | **Short-term (>1–4 weeks)** | Short training or educational series spanning more than one week up to four weeks. |
|  | **Medium-term (>4–12 weeks)** | Structured programs delivered over one to three months. |
|  | **Long-term (>12–24 weeks)** | Courses extending beyond one semester but under six months. |
|  | **Extended / Continuous (>24 weeks)** | Longitudinal or multi-semester integration within a curriculum. |
|  | **Unspecified** | Duration not provided or unclear. |
| **Facilitator Category** | **Faculty / Instructor** | University lecturers, professors, or course directors involved in formal teaching. |
|  | **Professional Staff (Non-clinical)** | University or program staff providing health promotion, academic advising, or non-clinical education. |
|  | **Licensed Mental Health Clinician** | Psychiatrists, psychologists, psychiatric nurses, or counselors formally trained in mental health care. |
|  | **Medical Clinician (Non-mental health)** | Physicians, nurses, midwives, or allied health clinicians not primarily specialized in mental health. |
|  | **Graduate / Teaching Assistant** | Postgraduate students or teaching assistants supporting course facilitation. |
|  | **Peer Facilitator (Student)** | Current students (e.g., peer mentors, RAs) leading or co-delivering components of the intervention. |
|  | **External Trainer / Consultant** | Guest experts or certified facilitators (e.g., Mental Health First Aid instructors). |
|  | **Community Partner / NGO** | Staff from community, nonprofit, or governmental organizations involved in delivery. |
|  | **Lived-experience Co-facilitator** | Individuals with personal experience of mental illness engaged in co-teaching or storytelling roles. |
|  | **Industry / Alumni Partner** | External professionals or graduates contributing through guest facilitation. |
|  | **Interdisciplinary Team (Mixed roles)** | Multi-professional teams co-facilitating (e.g., psychiatrist + nurse + peer educator). |
|  | **Self-guided / No human facilitator** | Automated, self-paced online modules or app-based learning without live instructor input. |
|  | **Not reported / Unknown** | Facilitator type not specified in the study. |
| **Outcome Domains** | **Knowledge** | Cognitive understanding or recall of mental health content, disorders, or treatments. |
|  | **Skills** | Ability to apply learned knowledge or perform relevant clinical/communication tasks. |
|  | **Self-efficacy & Confidence** | Belief in one’s capability to perform specific mental health–related tasks or support others. |
|  | **Attitudes & Stigma** | Beliefs, biases, or openness toward mental health, help-seeking, or persons with mental illness. |
|  | **Mental Health & Wellbeing** | Psychological or emotional functioning, stress levels, burnout, or resilience. |
|  | **Autonomy & Motivation** | Self-determination, motivation to act, or perceived agency in learning or helping. |
|  | **Satisfaction & Academic Achievement** | Learner satisfaction or performance outcomes such as grades or test scores. |
| **Assessor Category** | **Faculty-led Evaluation** | Evaluation conducted by course instructors or academic staff. |
|  | **Researcher-led Evaluation** | Assessment performed by independent or external research investigators. |
|  | **Program Facilitator Assessment** | Evaluation conducted by trainers or program implementers (non-faculty). |
|  | **Mixed Evaluation (Faculty & Researcher)** | Joint evaluation by teaching and research personnel. |
|  | **Student Self-assessment** | Participants assess their own knowledge, attitudes, or confidence. |
|  | **Institutional / Organizational Evaluation** | Conducted by external or internal committees for accreditation or quality assurance. |
|  | **Peer-led Evaluation** | Conducted by peers or colleagues providing feedback without faculty oversight. |
|  | **External Evaluation** | Conducted by independent consultants or third-party evaluators. |
|  | **Unspecified / Not reported** | No information provided on who assessed outcomes. |
| **Educational Setting** | **University classroom** | Traditional academic or lecture hall setting. |
|  | **Clinical placement** | Practical learning in hospitals, clinics, or patient care environments. |
|  | **Simulation lab** | Controlled simulated environment for experiential training. |
|  | **Online environment** | Virtual learning environment or learning management system. |
|  | **Hospital** | Hospital-based training not categorized as clinical placement. |
|  | **Community** | Community outreach, service-learning, or field-based education. |
|  | **Mixed** | Combination of two or more settings (e.g., classroom + clinical). |
| **Study Context** | **Single-site** | Conducted at one institution or setting. |
|  | **Two-site** | Conducted at two institutions or sites. |
|  | **Multi-site (National)** | Conducted across multiple sites within the same country. |
|  | **Multi-site (International)** | Conducted across institutions in different countries. |
| **Geographical Region** | **AFR, AMR, EMR, EUR, SEAR, WPR** | WHO regional classification; African Region, Region of the Americas, Eastern Mediterranean Region, European Region, South-East Asia Region, and Western Pacific Region. |
| **Kirkpatrick Level of Evaluation** | **Level 1 – Reaction** | Measures participants’ immediate responses to the educational intervention, including satisfaction, engagement, and perceived usefulness. Focuses on emotional and subjective reactions to learning. |
|  | **Level 2a – Learning (Attitudes)** | Captures changes in learners’ attitudes, beliefs, perceptions, or motivation following the intervention. Often reflects stigma reduction or attitudinal shifts toward mental health or help-seeking. |
|  | **Level 2b – Learning (Knowledge/Skills)** | Reflects gains in factual understanding, conceptual knowledge, or demonstrable skills as evidenced through tests, quizzes, or practical evaluations. |
|  | **Level 3 – Behavior** | Indicates application or transfer of learned knowledge and skills into practice, demonstrating observable or self-reported behavior change. |
|  | **Level 4 – Results (System/Organizational Impact)** | Represents broader impacts beyond the individual learner, including effects on organizational practices, clinical outcomes, policies, or curriculum reforms. |
| **Population groups** | **Uniprofessional** | Participants belong to a single health profession or discipline. |
|  | **Multi-professional** | Participants represent two or more distinct health professions or disciplines. |
| **Experiential Category** | **Role-play / Simulation** | Experiential learning through reenactment of clinical or interpersonal scenarios using simulated environments or actors to develop skills and decision-making. |
|  | **Reflective Exercises** | Structured activities prompting learners to analyse experiences, attitudes, or emotional responses to enhance insight and empathy. |
|  | **Real-world / Clinical Contact** | Direct engagement with real patients, communities, or authentic clinical environments where learners apply knowledge in practice. |
|  | **Mindfulness / Self-reflection** | Practices designed to enhance self-awareness, emotional regulation, and resilience through mindfulness-based techniques. |
|  | **Not reported** | No experiential learning component explicitly described in the study. |
| **Instructional Component** | **Didactic teaching / Lectures** | Formal instruction delivered through lectures, presentations, or structured teaching sessions aimed at transmitting knowledge. |
|  | **Group discussions / Peer learning** | Interactive learning through small or large group discussions, peer exchange, cooperative learning, or facilitated dialogue. |
|  | **Case-based learning / PBL** | Use of clinical or applied cases to support problem-based learning, clinical reasoning, and application of knowledge. |
|  | **Role-play / Simulation** | Experiential learning through simulated scenarios, role-playing, standardized patients, or virtual simulations to practice skills. |
|  | **Video / multimedia** | Use of audiovisual materials such as films, documentaries, video clips, animations, or motion graphics for instruction or reflection. |
|  | **Workshops / Interactive sessions** | Structured, hands-on sessions involving practical exercises, skills training, breakout activities, or guided practice. |
|  | **Mindfulness / Reflective activities** | Activities promoting self-awareness, emotional regulation, reflection, or wellbeing (e.g., mindfulness, meditation, reflective exercises). |
| **Technology Type** | **Simulation-based learning** | Involves the use of digital or virtual environments that mimic clinical scenarios for hands-on training, such as virtual patient simulators (e.g., vSim, INMEDEA). |
|  | **Video / Film materials** | Utilizes recorded audiovisual content for instruction, demonstration, or reflection (e.g., filmed patient encounters, instructional videos, documentaries). |
|  | **Online / e-learning platforms** | Refers to web-based learning management systems or structured online modules used for content delivery (e.g., Moodle, edX, EasyGenerator). |
|  | **Video conferencing tools** | Employs synchronous online communication technologies to facilitate remote teaching, discussions, or assessments (e.g., Zoom, Microsoft Teams, Google Meet). |
|  | **Surveys / interactive tools** | Involves digital tools enabling participation, feedback, or interactive learning (e.g., electronic surveys, polls, real-time quiz or voting systems). |
|  | **Not reported** | No technology-based tools were explicitly described in the study intervention. |
| **Evaluation Timing Category** | **Pre–Post Only** | Outcomes were assessed immediately before and immediately after the intervention, with no additional follow-up time point reported. |
|  | **Pre–Post + Follow-up** | Outcomes were assessed at baseline, immediately post-intervention, and at one or more follow-up time points after intervention completion. |
|  | **Not reported / Unclear** *(optional, if used)* | Evaluation timing was not sufficiently described to determine whether follow-up assessment occurred. |

**Supplementary 1C.** Included Studies

| **#** | **Study Design** | **DOI / URL** | **Article Information** |
| --- | --- | --- | --- |
| 1. | Qualitative | <https://doi.org/10.1007/s10447-023-09525-0> | Bamgbose, O. O., Aderonke Bamgbose Pederson, & Okocha, G. (2023). A Qualitative Study Exploring University Health Care Students Perceptions on Methods to Reduce Mental Health Stigma in Nigeria. *International Journal for the Advancement of Counselling*. https://doi.org/10.1007/s10447-023-09525-0 |
| 2. | Qualitative | <https://doi-org.uaeu.idm.oclc.org/10.4103/jpgm.jpgm_341_23> | Chen, Y., & Ting, C. (2023). Introduction of psychological skills laboratory in medical education. *Journal of Postgraduate Medicine*, *69*(4), 221–223. https://doi.org/10.4103/jpgm.jpgm_341_23 |
| 3. | Qualitative | <https://doi.org/10.1002/capr.12736> | Lucietto, C., Cristina, D., Dália Passos Sousa, Deise, Regina, M., & Fabio Scorsolini‐Comin. (2023). Online psychoeducation groups for mental health promotion: Perceptions of Brazilian university students. *Counselling and Psychotherapy Research*. https://doi.org/10.1002/capr.12736 |
| 4. | Qualitative | <https://doi.org/10.1080/01612840.2019.1631417> | Happell, B., Waks, S., Horgan, A., Greaney, S., Bocking, J., Manning, F., Goodwin, J., Scholz, B., Jan van der Vaart, K., Allon, J., Hals, E., Granerud, A., Doody, R., Wai-Chi Chan, S., Platania-Phung, C., Griffin, M., Russell, S., MacGabhann, L., Pulli, J., & Vatula, A. (2019). Expert by Experience Involvement in Mental Health Nursing Education: Nursing Students’ Perspectives on Potential Improvements. *Issues in Mental Health Nursing*, *40*(12), 1026–1033. https://doi.org/10.1080/01612840.2019.1631417 |
| 5. | Qualitative | <https://doi-org.uaeu.idm.oclc.org/10.1111/inm.12596> | Happell, B., Waks, S., Bocking, J., Horgan, A., Manning, F., Greaney, S., Goodwin, J., Scholz, B., Vaart, K. J., Allon, J., Granerud, A., Hals, E., Doody, R., Russell, S., Griffin, M., MacGabhann, L., Lahti, M., Ellilä, H., Pulli, J., & Vatula, A. (2019). “There’s more to a person than what’s in front of you”: Nursing students’ experiences of consumer taught mental health education. *International Journal of Mental Health Nursing*, *28*(4), 950–959. https://doi.org/10.1111/inm.12596 |
| 6. | Qualitative | <https://doi.org/10.1016/j.colegn.2019.02.006> | Hung, M. S. Y., Lam, S. K. K., & Chow, M. C. M. (2019). Nursing students’ experiences of mental health first aid training: A qualitative descriptive study. *Collegian*, *26*(5), 534–540. https://doi.org/10.1016/j.colegn.2019.02.006 |
| 7. | Qualitative | <https://www.nursingcenter.com/journalarticle?Article_ID=6498106&Journal_ID=3332683&Issue_ID=6497667> | *Sheridan, R., & Williams, J. (2021). Cinematic simulation. Nursing Education Perspectives, 43(6), 378–379. https://doi.org/10.1097/01.nep.0000000000000928* |
| 8. | Qualitative | <https://doi.org/10.3389/fmed.2023.1268277> | Sibéoni, J., Ellul, P., Théo Bubola, Yanis Debiche, & Piot, M.-A. (2023). Mental health first aid training among healthcare French students: a qualitative study. *Frontiers in Medicine*, *10*. https://doi.org/10.3389/fmed.2023.1268277 |
| 9. | Qualitative | <https://doi.org/10.1108/JMHTEP-03-2012-0002> | Wagstaff, C., Rose, J., Tew, J., Singh-Cooner, T., & Greening, J. (2013). Qualitative evaluation of learning between different professional groups. *The Journal of Mental Health Training, Education and Practice*, *8*(4), 219–228. https://doi.org/10.1108/jmhtep-03-2012-0002 |
| 10. | Qualitative | <http://dx.doi.org/10.1016/j.colegn.2016.04.004> | Kelly, J., & Birks, M. (2017). “ It’s the simple things you do first that start the process of help ”: Undergraduate nursing and midwifery students’ experiences of the Mental Health First Aid course. *Collegian*, *24*(3), 275–280. https://doi.org/10.1016/j.colegn.2016.04.004 |
| 11. | Mixed- Method | <https://doi.org/10.1186/s12909-022-03391-x> | Brown, M. E. L., MacLellan, A., Laughey, W., Omer, U., Himmi, G., LeBon, T., & Finn, G. M. (2022). Can stoic training develop medical student empathy and resilience? A mixed-methods study. *BMC Medical Education*, *22*(1). https://doi.org/10.1186/s12909-022-03391-x |
| 12. | Mixed- Method | <https://doi-org.uaeu.idm.oclc.org/10.1016/j.acap.2017.06.014> | Jee, S. H., Baldwin, C., Dadiz, R., Jones, M., & Alpert-Gillis, L. (2018). Integrated Mental Health Training for Pediatric and Psychology Trainees Using Standardized Patient Encounters. *Academic Pediatrics*, *18*(1), 119–121. https://doi.org/10.1016/j.acap.2017.06.014 |
| 13. | Mixed- Method | <http://dx.doi.org/10.1016/j.nepr.2016.06.004> | O’May, F., Gill, J., McWhirter, E., Kantartzis, S., Rees, C., & Murray, K. (2016). A teachable moment for the teachable moment? A prospective study to evaluate delivery of a workshop designed to increase knowledge and skills in relation to alcohol brief interventions (ABIs) amongst final year nursing and occupational therapy undergraduates. *Nurse Education in Practice*, *20*, 45–53. https://doi.org/10.1016/j.nepr.2016.06.004 |
| 14. | Mixed- Method | <https://doi.org/10.1016/j.ajpe.2023.100125> | Sevak, R. J., Chandler, C., Lui, M. C., Kaye, A. M., Halliwell, R. F., & Rogan, E. L. (2023). Impact of a Neuropsychiatric Therapeutics Course and a Case-Based Course on Pharmacy Students’ Mental Health Stigma. *American Journal of Pharmaceutical Education*, *87*(9), 100125–100125. https://doi.org/10.1016/j.ajpe.2023.100125 |
| 15. | Mixed- Method | <http://dx.doi.org/10.4314/ajpsy.v16i4.34> | Sheriff, R., Bass, N., Hughes, P., Ade-Odunlade, P., Ismail, A., Whitwell, S., & Jenkins, R. (2013). Use of interactive teaching techniques to introduce mental health training to medical schools in a resource poor setting. *African Journal of Psychiatry*, *16*(4). https://doi.org/10.4314/ajpsy.v16i4.34 |
| 16. | Observational | <https://pubmed-ncbi-nlm-nih-gov.uaeu.idm.oclc.org/10587771/> | Bethune, C., Worrall, G., D Freake, & Church, E. (1999). No psychiatry? Assessment of family medicine residents’ training in mental health issues. *PubMed*, *45*, 2636–2641. |
| 17. | Observational | <https://doi.org/10.1016/j.nedt.2021.105258> | Ferrara, P., Terzoni, S., Ruta, F., Poggi, A. D., Destrebecq, A., Gambini, O., & D’agostino, A. (2022). Nursing students’ attitudes towards suicide and suicidal patients: A multicentre cross-sectional survey. *Nurse Education Today*, *109*, 105258. https://doi.org/10.1016/j.nedt.2021.105258 |
| 18. | Observational | <https://doi.org/10.1371/journal.pone.0277525> | Herrmann-Werner, A., Erschens, R., Zipfel, S., & Festl-Wietek, T. (2022). Where there are challenges, there are opportunities: An undergraduate medical students’ teaching concept for mental health in times of COVID-19. *PLoS ONE*, *17*(11), e0277525. https://doi.org/10.1371/journal.pone.0277525 |
| 19. | Observational | <https://doi.org/10.1080/01612840.2020.1793248> | Liu, W. (2020). The Effects of Virtual Simulation on Undergraduate Nursing Students’ Mental Health Literacy: A Prospective Cohort Study. *Issues in Mental Health Nursing*, 1–10. https://doi.org/10.1080/01612840.2020.1793248 |
| 20. | Observational | <https://doi-org.uaeu.idm.oclc.org/10.1097/nne.0000000000001007> | Ozkara San, E., Dikec, G., Ata, E. E., & Sendir, M. (2021). Evaluation of the Use of Diverse Mental Health Simulation in Nursing Students’ Learning Experience. *Nurse Educator*, *Publish Ahead of Print*. https://doi.org/10.1097/nne.0000000000001007 |
| 21. | Observational | <https://doi.org/10.3390/ijerph19063213> | Giralt Palou, R., Prat Vigué, G., Romeu-Labayen, M., & Tort-Nasarre, G. (2022). Analysis of Stigma in Relation to Behaviour and Attitudes towards Mental Health as Influenced by Social Desirability in Nursing Students. *International Journal of Environmental Research and Public Health*, *19*(6), 3213. https://doi.org/10.3390/ijerph19063213 |
| 22. | Observational | <https://doi-org.uaeu.idm.oclc.org/10.1007/s11121-017-0840-y> | Patalay, P., Annis, J., Sharpe, H., Newman, R., Main, D., Ragunathan, T., Parkes, M., & Clarke, K. (2017). A Pre-Post Evaluation of OpenMinds: a Sustainable, Peer-Led Mental Health Literacy Programme in Universities and Secondary Schools. *Prevention Science*, *18*(8), 995–1005. https://doi.org/10.1007/s11121-017-0840-y |
| 23. | Observational | <https://doi-org.uaeu.idm.oclc.org/10.4103/1357-6283.178597> | Zimitat, C., De Silva, E., & Bowerman, L. (2015). A suicide awareness and intervention program for health professional students. *Education for Health*, *28*(3), 201. https://doi.org/10.4103/1357-6283.178597 |
| 24. | Randomized Controlled Trial | <https://doi.org/10.1007/s00787-022-02094-4> | Afsharnejad, B., Milbourn, B., Hayden-Evans, M., Baker-Young, E., Black, M. H., Thompson, C., McGarry, S., Grobler, M., Clifford, R., Zimmermann, F., Kacic, V., Hasking, P., Bölte, S., Romanos, M., Machingura, T., & Girdler, S. (2022). The efficacy of the “Talk-to-Me” suicide prevention and mental health education program for tertiary students: a crossover randomised control trial. *European Child & Adolescent Psychiatry*. https://doi.org/10.1007/s00787-022-02094-4 |
| 25. | Randomized Controlled Trial | <https://doi.org/10.1080/01421590600834252> | Bahar-Özvaris¸, S., Çetin, F. Ç., Turan, S., & Peters, A. S. (2006). Cooperative learning: a new application of problem-based learning in mental health training. *Medical Teacher*, *28*(6), 553–557. https://doi.org/10.1080/01421590600834252 |
| 26. | Randomized Controlled Trial | <https://doi.org/10.1186/s12888-017-1278-2> | Burns, S., Crawford, G., Hallett, J., Hunt, K., Chih, H. J., & Tilley, P. J. M. (2017). What’s wrong with John? a randomised controlled trial of Mental Health First Aid (MHFA) training with nursing students. *BMC Psychiatry*, *17*(1). https://doi.org/10.1186/s12888-017-1278-2 |
| 27. | Randomized Controlled Trial | <https://doi.org/10.1186/s12909-018-1154-x> | Davies, E. B., Beever, E., & Glazebrook, C. (2018). A pilot randomised controlled study of the Mental Health First Aid eLearning course with UK medical students. *BMC Medical Education*, *18*(1). https://doi.org/10.1186/s12909-018-1154-x |
| 28. | Randomized Controlled Trial | <http://www.biomedcentral.com/1472-6920/13/107> | de Vibe, M., Solhaug, I., Tyssen, R., Friborg, O., Rosenvinge, J. H., Sørlie, T., & Bjørndal, A. (2013). Mindfulness training for stress management: a randomised controlled study of medical and psychology students. *BMC Medical Education*, *13*(1). https://doi.org/10.1186/1472-6920-13-107 |
| 29. | Randomized Controlled Trial | <https://doi-org.uaeu.idm.oclc.org/10.1007/s40596-016-0592-1> | Fernandez, A., Tan, K.-A., Knaak, S., Chew, B. H., & Ghazali, S. S. (2016). Effects of Brief Psychoeducational Program on Stigma in Malaysian Pre-clinical Medical Students: A Randomized Controlled Trial. *Academic Psychiatry*, *40*(6), 905–911. https://doi.org/10.1007/s40596-016-0592-1 |
| 30. | Quasi-.Experimental | <https://doi.org/10.3138/ptc-2019-0036> | Edgar, S., & Connaughton, J. (2020). Using Mental Health First Aid Training to Improve the Mental Health Literacy of Physiotherapy Students. *Physiotherapy Canada*, *73*(2), e20190036. https://doi.org/10.3138/ptc-2019-0036 |
| 31. | Quasi-Experimental | <http://www.biomedcentral.com/1472-6920/11/51> | Kassam, A., Glozier, N., Leese, M., Loughran, J., & Thornicroft, G. (2011). A controlled trial of mental illness related stigma training for medical students. *BMC Medical Education*, *11*(1). https://doi.org/10.1186/1472-6920-11-51 |
| 32. | Quasi-Experimental | <https://doi-org.uaeu.idm.oclc.org/10.3109/00048674.2011.585454> | O’Reilly, C. L., Bell, J. S., Kelly, P. J., & Chen, T. F. (2011). Impact of Mental Health First aid Training on Pharmacy Students’ Knowledge, Attitudes and Self-Reported Behaviour: A Controlled Trial. *Australian & New Zealand Journal of Psychiatry*, *45*(7), 549–557. https://doi.org/10.3109/00048674.2011.585454 |
| 33. | Quasi-Experimental | <https://doi.org/10.1038/s41598-025-01633-8> | Alotaibi, N. M., Alenezi, L., Ahmad, F. M., Almutairi, W., Alshammari, D., Alenezi, M., & Alotaibi, M. (2025). A quasiexperimental study of assessing the impact of stress management program on health science students at Kuwait University. *Scientific Reports*, *15*(1). https://doi.org/10.1038/s41598-025-01633-8 |
| 34. | Quasi-Experimental | <https://doi.org/10.1007/s42399-022-01225-x> | Kotera, Y., Edwards, A.-M., Garip, G., Chircop, J., & Aledeh, M. (2022). Effects of Online Mental Health Classes on Mental Health and Stigma: a Controlled Before-After Study with 1-Month Follow-Up. *SN Comprehensive Clinical Medicine*, *4*(1). https://doi.org/10.1007/s42399-022-01225-x |
| 35. | Quasi-Experimental | <https://doi.org/10.1186/s12909-021-02990-4> | Kurki, M., Gilbert, S., Mishina, K., Lempinen, L., Luntamo, T., Hinkka-Yli-Salomäki, S., Sinokki, A., Upadhyaya, S., Wei, Y., & Sourander, A. (2021). Digital mental health literacy -program for the first-year medical students’ wellbeing: a one group quasi-experimental study. *BMC Medical Education*, *21*(1). https://doi.org/10.1186/s12909-021-02990-4 |
| 36. | Quasi-Experimental | <https://doi.org/10.3390/ijerph19095269> | Lai, H.-J., Lien, Y.-J., Chen, K.-R., & Lin, Y.-K. (2022). The Effectiveness of Mental Health Literacy Curriculum among Undergraduate Public Health Students. *International Journal of Environmental Research and Public Health*, *19*(9), 5269. https://doi.org/10.3390/ijerph19095269 |
| 37. | Quasi-Experimental | <https://doi.org/10.1186/s12909-022-03879-6> | Rodríguez-Ferrer, J. M., Manzano-León, A., Fernández-Jiménez, C., Aguilar-Parra, J. M., Cangas, A. J., & Luque de la Rosa, A. (2022). The use of digital escape rooms in nursing education. *BMC Medical Education*, *22*(1). https://doi.org/10.1186/s12909-022-03879-6 |
| 38. | Quasi-Experimenta | <https://doi.org/10.1016/j.apnu.2025.01.005> | Selim, A., Newby, C., Almutairy, A., Aldossari, A., Alkabba, F., Arabi, S., Zoromba, M. A., Atta, M. H. R., & Ibrahim, N. (2025). Enhancing warning signs of mental health literacy: Evaluating a digital base intervention for health profession students. *Archives of Psychiatric Nursing*, *54*, 91–101. https://doi.org/10.1016/j.apnu.2025.01.005 |
| 39. | Quasi-Experimental | <https://doi.org/10.12688/f1000research.111002.2> | Asumi, T., Hirokazu, T., Ayumi, T., Takami, M., Yuki, S., Asaki, M., & Tetsuaki, A. (2023). Crisis-management, Anti-stigma, and Mental Health Literacy Program for University Students (CAMPUS): A preliminary evaluation of suicide prevention. *ProQuest*. https://doi.org/10.12688/f1000research.111002.2 |
| 40. | Quasi-Experimental | <https://doi-org.uaeu.idm.oclc.org/10.1080/08897077.2011.640151> | Tanner, T. B., Wilhelm, S. E., Rossie, K. M., & Metcalf, M. P. (2012). Web-Based SBIRT Skills Training for Health Professional Students and Primary Care Providers. *Substance Abuse*, *33*(3), 316–320. https://doi.org/10.1080/08897077.2011.640151 |
| 41. | Quasi-Experimental | <https://doi.org/10.1186/s12909-023-05019-0> | Till, B., Hofhansl, A., & Niederkrotenthaler, T. (2024). Effects of the mental health promotion seminar “Coping with stress” in the undergraduate medical curriculum of the Medical University of Vienna. *BMC Medical Education*, *24*(1). https://doi.org/10.1186/s12909-023-05019-0 |
| 42. | Quasi-Experimental | <https://doi.org/10.4088/PCC.17m02210> | Ureste, P. J., Duong, T. L., Seritan, A. L., Iosif, A.-M., & Hilty, D. M. (2017). Improving Mental Health Training for Primary Care Residents. *The Primary Care Companion for CNS Disorders*, *19*(5). https://doi.org/10.4088/pcc.17m02210 |
| 43. | Quasi-Experimental | <https://doi.org/10.3390/nursrep14040216> | Valentim, O., Correia, T., Moutinho, L., Seabra, P., Querido, A., & Laranjeira, C. (2024). “This Is Me” an Awareness-Raising and Anti-Stigma Program for Undergraduate Nursing Students: A Pre-Post Intervention Study. *Nursing Reports*, *14*(4), 2956–2974. https://doi.org/10.3390/nursrep14040216 |
| 44. | Quasi-Experimental | <https://doi.org/10.1186/s12909-020-02438-1> | Wang, Q., & Du, T. (2020). Implementation of the college student mental health education course (CSMHEC) in undergraduate medical curriculum: effects and insights. *BMC Medical Education*, *20*(1). https://doi.org/10.1186/s12909-020-02438-1 |
| 45. | Quasi-Experimental | <https://doi-org.uaeu.idm.oclc.org/10.1097/01.nep.0000000000000796> | Abuhammad, S., & Hamaideh, S. (2021). Nursing Students’ Attitudes Toward Seeking Professional Psychological Help Before and After Attending a Mental Health Course. *Nursing Education Perspectives*, *Publish Ahead of Print*. https://doi.org/10.1097/01.nep.0000000000000796 |
| 46. | Quasi-Experimental | [https://doi.org/10.1371/journal. pone.0319431](https://doi.org/10.1371/journal.%20pone.0319431) | Agudelo-Hernández, F., Vélez-Botero, H., & Guapacha-Montoya, M. (2025). Results of quality rights on human rights engagement, stigma and attitudes towards mental health among Colombian medical students. *PLoS ONE*, *20*(2), e0319431. https://doi.org/10.1371/journal.pone.0319431 |
| 47. | Quasi-Experimental | <https://doi-org.uaeu.idm.oclc.org/10.1186/s40359-015-0069-0> | Bond, K. S., Jorm, A. F., Kitchener, B. A., & Reavley, N. J. (2015). Mental health first aid training for Australian medical and nursing students: an evaluation study. *BMC Psychology*, *3*(1). https://doi.org/10.1186/s40359-015-0069-0 |
| 48. | Quasi-Experimental | <https://doi-org.uaeu.idm.oclc.org/10.1111/j.1365-2850.2009.01421.x> | Brown, J. F. (2009). Faith‐based mental health education: a service‐learning opportunity for nursing students. *Journal of Psychiatric and Mental Health Nursing*, *16*(6), 581–588. https://doi.org/10.1111/j.1365-2850.2009.01421.x |
| 49. | Quasi-Experimental | <https://doi-org.uaeu.idm.oclc.org/10.1097/01.nep.0000000000001303> | Gamez, V., & Gunning, T. (2024). Improving Mental Health Literacy in Nursing Students: Integrating Mental Health Throughout the Curriculum. *Nursing Education Perspectives*, *45*(5), 298–300. https://doi.org/10.1097/01.nep.0000000000001303 |
| 50. | Quasi-Experimental | <https://doi.org/10.1016/j.nepr.2021.103009> | Gu, L., Jiao, W., Xia, H., & Yu, M. (2021). Psychiatric-mental health education with integrated role-play and real-world contact can reduce the stigma of nursing students towards people with mental illness. *Nurse Education in Practice*, *52*, 103009. https://doi.org/10.1016/j.nepr.2021.103009 |
| 51. | Quasi-Experimental | <http://dx.doi.org/10.1016/j.nedt.2015.09.007> | Higgins, A., Carroll, M., & Sharek, D. (2016). Impact of perinatal mental health education on student midwives’ knowledge, skills and attitudes: A pre/post evaluation of a module of study. *Nurse Education Today*, *36*, 364–369. https://doi.org/10.1016/j.nedt.2015.09.007 |
| 52. | Quasi-Experimental | https://doi.org/10.12707/RIV18087 | Loureiro, L., & Costa, L. (2019b). Evaluation of the Mental Health First Aid program in undergraduate nursing students. *Revista De Enfermagem Referência*, *IV Série*(N^o^ 20), 9–18. https://doi.org/10.12707/riv18087 |
| 53. | Quasi-Experimental | <https://doi.org/10.1007/s00737-023-01386-1> | Marty, L., Myrick, O., Perelman, A., Kotlyar, A., & Vernon, J. (2023). Filling a gap in OBGYN education: a pilot lecture series on perinatal mental health. *Archives of Women’s Mental Health*. https://doi.org/10.1007/s00737-023-01386-1 |
| 54. | Quasi-Experimental | <https://doi-org.uaeu.idm.oclc.org/10.1080/08897077.2011.640103> | Satterfield, J. M., O’Sullivan, P., Satre, D. D., Tsoh, J. Y., Batki, S. L., Julian, K., McCance-Katz, E. F., & Wamsley, M. (2012). Using Standardized Patients to Evaluate Screening, Brief Intervention, and Referral to Treatment (SBIRT) Knowledge and Skill Acquisition for Internal Medicine Residents. *Substance Abuse*, *33*(3), 303–307. https://doi.org/10.1080/08897077.2011.640103 |
| 55. | Quasi-Experimental | <https://doi.org/10.5812/ijpbs.9672.> | Vaghee, S., Salarhaji, A., Kashani Lotfabadi, M., & Vaghei, N. (2017). Comparing the Effect of Contact-based Education with Acceptance and Commitment Training on Destigmatization Toward Psychiatric Disorders in Nursing Students. *Iranian Journal of Psychiatry and Behavioral Sciences*, *11*(2). https://doi.org/10.5812/ijpbs.9672 |

**Supplementary 1 D. Study design and outcome characteristics by WHO region**

**Table S1D1. Measured outcome domains by WHO region**

| **WHO Region** | **Outcome** | **count** |
| --- | --- | --- |
| African Region (AFR) | Attitudes & Stigma | 1 |
|  | Knowledge | 1 |
| Eastern Mediterranean Region (EMR) | Attitudes & Stigma | 2 |
|  | Knowledge | 1 |
|  | Mental Health & Wellbeing | 1 |
| European Region (EUR) | Knowledge | 11 |
|  | Attitudes & Stigma | 4 |
|  | Skills | 2 |
|  | Mental Health & Wellbeing | 1 |
|  | Self-Efficacy & Confidence | 1 |
| Region of the Americas (AMR) | Knowledge | 7 |
|  | Self-Efficacy & Confidence | 3 |
|  | Attitudes & Stigma | 2 |
|  | Skills | 2 |
| Western Pacific Region (WPR) | Knowledge | 6 |
|  | Attitudes & Stigma | 4 |
|  | Self-Efficacy & Confidence | 3 |
|  | Skills | 2 |
|  | Mental Health & Wellbeing | 1 |

**Figure S1D1. Distribution of measured outcome domains by WHO region**
Stacked bar chart showing the number of studies reporting each outcome domain across WHO regions. Studies may contribute to multiple outcome categories.

**Table S1D2. Study design distribution by WHO region**

| **Design Group** | **African Region (AFR)** | **Eastern Mediterranean Region (EMR)** | **European Region (EUR)** | **Region of the Americas (AMR)** | **Western Pacific Region (WPR)** |
| --- | --- | --- | --- | --- | --- |
| **RCT** | 0 | 0 | 3 | 0 | 3 |
| **Quasi** | 0 | 4 | 8 | 7 | 7 |
| **Mixed methods** | 1 | 0 | 2 | 2 | 0 |
| **Observational** | 0 | 0 | 4 | 3 | 1 |
| **Qualitative** | 1 | 0 | 2 | 2 | 5 |

**Figure S1D2. Study design groups by WHO region**
Heatmap illustrating the distribution of study design groups across WHO regions, with darker shading indicating higher study counts.

**Supplementary 1E.**Temporal trends in the adoption of experiential components and educational technology

**Table S1E** Yearly counts and cumulative adoption of experiential components and technology use across included studies (1999–2025).

| **Year** | **n_studies** | **n_experiential** | **n_tech** | **cum_experiential** | **cum_tech** |
| --- | --- | --- | --- | --- | --- |
| 1999 | 1 | 1 | 0 | 1 | 0 |
| 2006 | 1 | 1 | 0 | 2 | 0 |
| 2009 | 1 | 1 | 1 | 3 | 1 |
| 2011 | 2 | 2 | 1 | 5 | 2 |
| 2012 | 2 | 2 | 2 | 7 | 4 |
| 2013 | 3 | 3 | 2 | 10 | 6 |
| 2015 | 2 | 2 | 2 | 12 | 8 |
| 2016 | 3 | 3 | 2 | 15 | 10 |
| 2017 | 5 | 5 | 2 | 20 | 12 |
| 2018 | 2 | 2 | 2 | 22 | 14 |
| 2019 | 4 | 4 | 0 | 26 | 14 |
| 2020 | 1 | 1 | 1 | 27 | 15 |
| 2021 | 6 | 6 | 4 | 33 | 19 |
| 2022 | 8 | 8 | 6 | 41 | 25 |
| 2023 | 7 | 7 | 7 | 48 | 32 |
| 2024 | 4 | 4 | 2 | 52 | 34 |
| 2025 | 2 | 2 | 1 | 54 | 35 |
| **Note.** n_studies=number of studies published in that year; n_experiential=number of studies reporting at least one experiential component; n_tech=number of studies using educational technology; cum_experiential=cumulative experiential studies; cum_tech=cumulative technology studies. | | | | | |

**Figure S1E. Cumulative adoption over time of experiential components and educational technology in health‑professional training (1999–2025).**
The green step line shows the cumulative number of studies that included any **experiential component**; the blue step line shows the cumulative number of studies that used any **technology**. Numeric labels mark cumulative totals at each year. Steeper segments indicate periods of accelerated uptake.

**Supplementary 1F.** Sample size distribution by study design

| Design type | Min sample size | Median sample size | Max sample size |
| --- | --- | --- | --- |
| Quasi-experimental | 15 | 110 | 493 |
| Randomised controlled trials (RCTs) | 55 | 129 | 288 |
| Mixed methods | 24 | 50 | 202 |
| Observational | 62 | 160 | 314 |

**Supplementary 1G.** Theoretical frameworks and models applied in mental health literacy educational interventions for health professions students, categorized by framework type

| Framework Type | Theoretical Framework / Model Used | Study IDs |
| --- | --- | --- |
| Mental Health Literacy & Stigma | Jorm’s (2000) Mental Health Literacy framework | [1], [2], [3], |
|  | MHFA framework (ALGEE) | [4] |
|  | PERMA framework (mental fitness, resilience) | [5], [6] |
| Psychological & Therapeutic Models | Cognitive Behavioral Therapy (CBT) | [7], [8] |
|  | Motivational Interviewing (MI), FRAMES model | [9] |
|  | Mindfulness-Based Stress Reduction (MBSR) | [10], [7] |
|  | Stoic Philosophy (Negative visualisation, Stoic mindfulness, Stoic reflection) | [11] |
|  | Ladder of Involvement (Arnstein, 1969; Tew et al., 2004) | [12] |
| Educational & Curriculum Models | Kern’s six-step approach to curriculum development | [1] |
|  | Knowledge-Attitude-Practice (KAP) model | [13] |
|  | Student Team Learning (STL) (Slavin, 1994) | [14] |
|  | NLN Jeffries Simulation Theory, INACSL Standards | [15] |
|  | Jeffreys’ Cultural Competence and Confidence (CCC) model; ASPE, INACSL Standards | [16] |
|  | CAPE 2013 domains | [17] |
| Community Models | Community-based SAIP model; ABC model (Ask, Behaviours, Current plan, Discover) | [18] |

**Supplementary 1H.** Topic Coverage Across Included Studies

| Topic | Frequency (n) | Representative Focus |
| --- | --- | --- |
| Depression | 42 | Recognition, management, education, and stigma reduction |
| Anxiety disorders | 39 | Recognition, coping, and crisis management |
| Suicide prevention / crisis response | 34 | Risk assessment, gatekeeper training, and help-seeking |
| Substance use disorders | 22 | Screening, brief intervention, and referral (SBIRT) |
| Psychosis / schizophrenia | 18 | Recognition, communication, and stigma reduction |
| Stress management | 17 | Coping, resilience, mindfulness, burnout prevention |
| Stigma and discrimination | 16 | Attitude change, empathy, and recovery-oriented practice |
| Communication skills | 14 | Therapeutic communication, active listening, breaking bad news |
| Recovery-oriented / person-centred care | 12 | Holistic and consumer-inclusive practice |
| Mindfulness and self-care | 10 | Relaxation, metacognition, and emotion regulation |
| Family and relationship dynamics | 8 | Family influence, support networks, and relational self-care |
| Human rights / social inclusion | 6 | Advocacy, empowerment, and disability inclusion |
| Trauma and PTSD | 5 | Awareness, prevention, and support |
| Eating disorders | 4 | Recognition and treatment guidance |
| Maternal/perinatal mental health | 3 | Screening tools, pharmacological management, support services |
| Other/interdisciplinary themes | 9 | Professional ethics, spirituality, workplace wellbeing |

**Supplementary 1I.** List of Assessment Scales and Instruments used in mental health literacy educational interventions for health professions students, categorized by scale category

| **Scale Category** | **Scale names** |
| --- | --- |
| **Attitudes, Stigma & Beliefs** | - Opening Minds Stigma Scale for HealthCare Providers (OMS-HC)- [19], [10], [17] - Community Attitudes toward the Mentally Ill (CAMI) - [20] - Mental Illness Clinicians’ Attitudes Scale (MICA / MICA-4) -[21], [22],[22],[23],[22],[20] - Attribution Questionnaire (AQ-9 /AQ-27)-[22],[24], [17], - Social Distance Scale (SDS)-[25],[5], [4] - Mental Health Literacy Scale (MHLS)- [26],[27], - Mental Health Knowledge Schedule (MAKS)- [22] - Attitudes Toward Psychiatry and Mental Health (ATP-30)-[28] - Mental Health Literacy Scale for Healthcare Students (MHLS-HS)-[29],[20] - Questionnaire on Public Stigma Toward Mental Disorder (AQ-14/AQ-9)-[30],[17],[22] - Jefferson Scale of Empathy (JSE)-[22],[11] - Stigma towards People with Mental Illness Scale (SPMI)-[31] - Attitudes towards Women with Mental Illness -[32] - Depression Stigma Scale (DSS)-[5],[33] - Stoic Attitudes and Behaviours Scale (SABS)-[11] - QuALiSMental (Questionnaire for Assessment of Mental Health Literacy) -[34] - Short form of the Attribution Questionnaire, Italian version-[35] - Scale for the Measurement of Attitudes Toward Mental Illness and Training in Mental Health Disorders-[23] - Reported and Intended Behaviour Scale (RIBS)-[23] - Interpersonal Attitudes Scale(IAS)-[22] - Level of Familiarity Scale(LOF)-[27] |
| **Suicide Prevention & Crisis Response** | - Suicide Intervention Response Inventory (SIRI-2)-[36] - Gatekeeper Self-Efficacy Scale (GKSES)-[24] - Suicidal Behaviors Questionnaire–Revised (SBQ-R)-[24] |
| **Help-Seeking & Professional Attitudes** | - General Help-Seeking Questionnaire (GHSQ)-[24],[10] - Attitudes Toward Seeking Professional Psychological Help Scale (ATSPPHS / ATSPPHS-SF)-[37],[38] - Help-Seeking Intentions (GHSQ) -[24] - Helping Attitudes Scale (HAS)-[4] |
| **Resilience, Empowerment & Recovery** | - Brief Resilience Scale (BRS)-[38],[11] - Empowerment Scale -[17] - Recovery Scale -[17] - Perceived Medical School Stress Scale (PMSS)-[10] - Perception of Academic Stress Scale (PASS) -[36] |
| **Stress, Burnout & Well-being** | - Burnout Assessment Tool (BAT)-[39] - Chinese College Student Academic Burnout Inventory (CCSABI)-[40] - Maslach Burnout Inventory (MBI / MBI-SS)-[10] - Perceived Stress Scale (PSS-10)-[41], [6] - Depression, Anxiety, and Stress Scale (DASS-21)-[41], [42],[40] - Stress and Coping Inventory (Physical and Psychological Stress Subscale)-[39] - Five Facet Mindfulness Questionnaire (FFMQ)-[10] - Satisfaction With Life Scale (SWLS)-[39],[40] - General Health Questionnaire (GHQ-12)-[6] - Self-Reflection and Insight Scale (SRIS)-[39] - PHQ-9 -[24] - Intergroup Anxiety Scale (IAS) -[22] - Subjective Well-Being Scale (SWB)-[10] |
| **Self-Efficacy, Empathy & Communication** | - General Self-Efficacy Scale (GSE)-[36] - Interpersonal Attraction Scale (IAS)- [43] - Communication Assessment Tool (CAT)-[43] - Motivational Interviewing Competency Assessment (MI Skills Assessment)- [43] - Self-Acceptance Scale (Tsukawaki et al., 2009)-[24] - Japanese Self-Concealment Scale (JSCS)-[24] |
| **Learning, Knowledge & Simulation Evaluation** | - Observed Structured Clinical Examination (OSCE)-[44] - Objective Structured Video Examination (OSVE)-[36], [9] - Simulation Effectiveness Tool / SET-M (Modified)-[9],[44] - Student Satisfaction and Self-Confidence in Learning Scale (NLN Instrument)-[44] - Mental Health First Aid Course Evaluation /Knowledge Test (MHFA Evaluation Form / MHFA Quiz)-[2],[5],[33] |
| **Reflection, Professional Growth** | - Reflective Practice Questionnaire (RPQ)-[11] - Personality Reflection Tool (Self-Report Reflection Inventory)-[11] - Reflection Logs / Journals-[9], [44] |
| **Satisfaction** | - Client Satisfaction Questionnaire (CSQ-I)-[6] - HRXSW – Human Rights Exposure Scale for Workers -[20] - HRESW – Human Rights Engagement Scale for Workers-[20] |

**Supplementary 1J.** Evaluation Timing and Follow-up by Study Design

| Study Design | Pre–Post Only | Pre–Post + Follow-up |
| --- | --- | --- |
| RCT Design | 3 | 3 |
| Quasi-Experimental Design | 20 | 6 |
| Total | 23 | 9 |

**Supplementary 1K.** Summary of Assessment Approaches

| **Assessor Type** | **Studies *(n)*** | **Description / Notes** |
| --- | --- | --- |
| Student self-assessment | 41 | Predominant method. Included self-reported pre/post surveys, questionnaires, and self-reflection exercises assessing knowledge, attitudes, or confidence. |
| Faculty/instructor assessment | 7 | Faculty-graded components such as OSCEs, simulation debriefings, or evaluation of reflective journals. Some used blinded assessors for objectivity. |
| Researcher assessment | 1 | Researchers analyzed qualitative data (e.g., reflective narratives) to identify themes related to learning impact. |
| Peer assessment | 3 | Limited use in simulation and small-group settings, where students rated peers using rubrics or feedback checklists. |
| External/expert evaluators | 2 | External raters or standardized examiners (e.g., OSCE evaluators) are used for objective scoring. |
| Mixed assessment | 1 | Few studies have triangulated self-, peer-, and faculty perspectives for comprehensive evaluation. |

**References**

[1]	A. Herrmann-Werner, R. Erschens, S. Zipfel, and T. Festl-Wietek, “Where there are challenges, there are opportunities: An undergraduate medical students’ teaching concept for mental health in times of COVID-19,” *PLoS ONE*, vol. 17, no. 11, p. e0277525, Nov. 2022, doi: 10.1371/journal.pone.0277525.

[2]	K. S. Bond, A. F. Jorm, B. A. Kitchener, and N. J. Reavley, “Mental health first aid training for Australian medical and nursing students: an evaluation study,” *BMC Psychol*, vol. 3, no. 1, p. 11, Dec. 2015, doi: 10.1186/s40359-015-0069-0.

[3]	M. S. Y. Hung, S. K. K. Lam, and M. C. M. Chow, “Nursing students’ experiences of mental health first aid training: A qualitative descriptive study,” *Collegian*, vol. 26, no. 5, pp. 534–540, Oct. 2019, doi: 10.1016/j.colegn.2019.02.006.

[4]	P. Patalay *et al.*, “A Pre-Post Evaluation of OpenMinds: a Sustainable, Peer-Led Mental Health Literacy Programme in Universities and Secondary Schools,” *Prev Sci*, vol. 18, no. 8, pp. 995–1005, Nov. 2017, doi: 10.1007/s11121-017-0840-y.

[5]	S. Burns, G. Crawford, J. Hallett, K. Hunt, H. J. Chih, and P. J. M. Tilley, “What’s wrong with John? a randomised controlled trial of Mental Health First Aid (MHFA) training with nursing students,” *BMC Psychiatry*, vol. 17, no. 1, p. 111, Dec. 2017, doi: 10.1186/s12888-017-1278-2.

[6]	M. Kurki *et al.*, “Digital mental health literacy -program for the first-year medical students’ wellbeing: a one group quasi-experimental study,” *BMC Med Educ*, vol. 21, no. 1, p. 563, Dec. 2021, doi: 10.1186/s12909-021-02990-4.

[7]	Y. Chen and C. Ting, “Introduction of psychological skills laboratory in medical education,” *Journal of Postgraduate Medicine*, vol. 69, no. 4, pp. 221–223, Oct. 2023, doi: 10.4103/jpgm.jpgm_341_23.

[8]	G. C. L. Da Silva, D. C. D. S. Pereira, D. P. Sousa, D. C. De Souza, M. R. G. Correia‐Zanini, and F. Scorsolini‐Comin, “Online psychoeducation groups for mental health promotion: Perceptions of Brazilian university students,” *Couns and Psychother Res*, vol. 24, no. 4, pp. 1232–1242, Dec. 2024, doi: 10.1002/capr.12736.

[9]	F. O’May, J. Gill, E. McWhirter, S. Kantartzis, C. Rees, and K. Murray, “A teachable moment for the teachable moment? A prospective study to evaluate delivery of a workshop designed to increase knowledge and skills in relation to alcohol brief interventions (ABIs) amongst final year nursing and occupational therapy undergraduates,” *Nurse Education in Practice*, vol. 20, pp. 45–53, Sep. 2016, doi: 10.1016/j.nepr.2016.06.004.

[10]	M. De Vibe *et al.*, “Mindfulness training for stress management: a randomised controlled study of medical and psychology students,” *BMC Med Educ*, vol. 13, no. 1, p. 107, Dec. 2013, doi: 10.1186/1472-6920-13-107.

[11]	M. E. L. Brown *et al.*, “Can stoic training develop medical student empathy and resilience? A mixed-methods study,” *BMC Med Educ*, vol. 22, no. 1, p. 340, Dec. 2022, doi: 10.1186/s12909-022-03391-x.

[12]	C. Wagstaff, J. Rose, J. Tew, T. Singh-Cooner, and J. Greening, “Qualitative evaluation of learning between different professional groups,” *The Journal of Mental Health Training, Education and Practice*, vol. 8, no. 4, pp. 219–228, Nov. 2013, doi: 10.1108/JMHTEP-03-2012-0002.

[13]	L. Gu, W. Jiao, H. Xia, and M. Yu, “Psychiatric-mental health education with integrated role-play and real-world contact can reduce the stigma of nursing students towards people with mental illness,” *Nurse Education in Practice*, vol. 52, p. 103009, Mar. 2021, doi: 10.1016/j.nepr.2021.103009.

[14]	S. Bahar-Özvaris¸, F. Ç. Çetin, S. Turan, and A. S. Peters, “Cooperative learning: a new application of problem-based learning in mental health training,” *Medical Teacher*, vol. 28, no. 6, pp. 553–557, Jan. 2006, doi: 10.1080/01421590600834252.

[15]	R. Sheridan and J. Williams, “Cinematic Simulation: An Innovative Approach for Teaching Psychiatric Mental Health Nursing,” *Nurs Educ Perspect*, vol. 43, no. 6, pp. 378–379, Nov. 2022, doi: 10.1097/01.NEP.0000000000000928.

[16]	E. Ozkara San, G. Dikec, E. E. Ata, and M. Sendir, “Evaluation of the Use of Diverse Mental Health Simulation in Nursing Students’ Learning Experience: An International Multisite Study,” *Nurse Educ*, vol. 46, no. 6, pp. E148–E153, Nov. 2021, doi: 10.1097/NNE.0000000000001007.

[17]	R. J. Sevak, C. Chandler, M. C. Lui, A. M. Kaye, R. F. Halliwell, and E. L. Rogan, “Impact of a Neuropsychiatric Therapeutics Course and a Case-Based Course on Pharmacy Students’ Mental Health Stigma,” *American Journal of Pharmaceutical Education*, vol. 87, no. 9, p. 100125, Sep. 2023, doi: 10.1016/j.ajpe.2023.100125.

[18]	E. De Silva, L. Bowerman, and C. Zimitat, “A suicide awareness and intervention program for health professional students,” *Educ Health*, vol. 28, no. 3, p. 201, 2015, doi: 10.4103/1357-6283.178597.

[19]	S. Vaghee, A. Salarhaji, M. Kashani Lotfabadi, and N. Vaghei, “Comparing the Effect of Contact-based Education with Acceptance and Commitment Training on Destigmatization Toward Psychiatric Disorders in Nursing Students,” *Iran J Psychiatry Behav Sci*, vol. 11, no. 2, Mar. 2017, doi: 10.5812/ijpbs.9672.

[20]	F. Agudelo-Hernández, H. Vélez-Botero, and M. Guapacha-Montoya, “Results of quality rights on human rights engagement, stigma and attitudes towards mental health among Colombian medical students,” *PLoS ONE*, vol. 20, no. 2, p. e0319431, Feb. 2025, doi: 10.1371/journal.pone.0319431.

[21]	A. Kassam, N. Glozier, M. Leese, J. Loughran, and G. Thornicroft, “A controlled trial of mental illness related stigma training for medical students,” *BMC Med Educ*, vol. 11, no. 1, p. 51, Dec. 2011, doi: 10.1186/1472-6920-11-51.

[22]	O. Valentim, T. Correia, L. Moutinho, P. Seabra, A. Querido, and C. Laranjeira, “‘This Is Me’ an Awareness-Raising and Anti-Stigma Program for Undergraduate Nursing Students: A Pre-Post Intervention Study,” *Nursing Reports*, vol. 14, no. 4, pp. 2956–2974, Oct. 2024, doi: 10.3390/nursrep14040216.

[23]	R. Giralt Palou, G. Prat Vigué, M. Romeu-Labayen, and G. Tort-Nasarre, “Analysis of Stigma in Relation to Behaviour and Attitudes towards Mental Health as Influenced by Social Desirability in Nursing Students,” *IJERPH*, vol. 19, no. 6, p. 3213, Mar. 2022, doi: 10.3390/ijerph19063213.

[24]	A. Takahashi *et al.*, “preliminary evaluation of suicide prevention,” 2023.

[25]	C. L. O’Reilly, J. S. Bell, P. J. Kelly, and T. F. Chen, “Impact of Mental Health First aid Training on Pharmacy Students’ Knowledge, Attitudes and Self-Reported Behaviour: A Controlled Trial,” *Aust N Z J Psychiatry*, vol. 45, no. 7, pp. 549–557, Jul. 2011, doi: 10.3109/00048674.2011.585454.

[26]	A. Selim *et al.*, “Enhancing warning signs of mental health literacy: Evaluating a digital base intervention for health profession students,” *Archives of Psychiatric Nursing*, vol. 54, pp. 91–101, Feb. 2025, doi: 10.1016/j.apnu.2025.01.005.

[27]	J. F. Brown, “Faith‐based mental health education: a service‐learning opportunity for nursing students,” *Psychiatric Ment Health Nurs*, vol. 16, no. 6, pp. 581–588, Aug. 2009, doi: 10.1111/j.1365-2850.2009.01421.x.

[28]	S. Edgar and J. Connaughton, “Using Mental Health First Aid Training to Improve the Mental Health Literacy of Physiotherapy Students,” *Physiotherapy Canada*, vol. 73, no. 2, pp. 188–193, Apr. 2021, doi: 10.3138/ptc-2019-0036.

[29]	H.-J. Lai, Y.-J. Lien, K.-R. Chen, and Y.-K. Lin, “The Effectiveness of Mental Health Literacy Curriculum among Undergraduate Public Health Students,” *IJERPH*, vol. 19, no. 9, p. 5269, Apr. 2022, doi: 10.3390/ijerph19095269.

[30]	J. M. Rodríguez-Ferrer, A. Manzano-León, C. Fernández-Jiménez, J. M. Aguilar-Parra, A. J. Cangas, and A. Luque De La Rosa, “The use of digital escape rooms in nursing education,” *BMC Med Educ*, vol. 22, no. 1, p. 901, Dec. 2022, doi: 10.1186/s12909-022-03879-6.

[31]	L. Gu, W. Jiao, H. Xia, and M. Yu, “Psychiatric-mental health education with integrated role-play and real-world contact can reduce the stigma of nursing students towards people with mental illness,” *Nurse Education in Practice*, vol. 52, p. 103009, Mar. 2021, doi: 10.1016/j.nepr.2021.103009.

[32]	A. Higgins, M. Carroll, and D. Sharek, “Impact of perinatal mental health education on student midwives’ knowledge, skills and attitudes: A pre/post evaluation of a module of study,” *Nurse Education Today*, vol. 36, pp. 364–369, Jan. 2016, doi: 10.1016/j.nedt.2015.09.007.

[33]	E. B. Davies, E. Beever, and C. Glazebrook, “A pilot randomised controlled study of the Mental Health First Aid eLearning course with UK medical students,” *BMC Med Educ*, vol. 18, no. 1, p. 45, Dec. 2018, doi: 10.1186/s12909-018-1154-x.

[34]	L. Loureiro and L. Costa, “Evaluation of the Mental Health First Aid program in undergraduate nursing students,” *Rev. Enf. Ref.*, vol. IV Série, no. N^o^ 20, pp. 9–18, Mar. 2019, doi: 10.12707/RIV18087.

[35]	P. Ferrara *et al.*, “Nursing students’ attitudes towards suicide and suicidal patients: A multicentre cross-sectional survey,” *Nurse Education Today*, vol. 109, p. 105258, Feb. 2022, doi: 10.1016/j.nedt.2021.105258.

[36]	B. Afsharnejad *et al.*, “The efficacy of the ‘Talk-to-Me’ suicide prevention and mental health education program for tertiary students: a crossover randomised control trial,” *Eur Child Adolesc Psychiatry*, vol. 32, no. 12, pp. 2477–2489, Dec. 2023, doi: 10.1007/s00787-022-02094-4.

[37]	S. Abuhammad and S. Hamaideh, “Nursing Students’ Attitudes Toward Seeking Professional Psychological Help Before and After Attending a Mental Health Course,” *Nurs Educ Perspect*, vol. 43, no. 2, pp. 129–131, Mar. 2022, doi: 10.1097/01.NEP.0000000000000796.

[38]	B. Afsharnejad *et al.*, “The efficacy of the ‘Talk-to-Me’ suicide prevention and mental health education program for tertiary students: a crossover randomised control trial,” *Eur Child Adolesc Psychiatry*, vol. 32, no. 12, pp. 2477–2489, Dec. 2023, doi: 10.1007/s00787-022-02094-4.

[39]	B. Till, A. Hofhansl, and T. Niederkrotenthaler, “Effects of the mental health promotion seminar ‘Coping with stress’ in the undergraduate medical curriculum of the Medical University of Vienna,” *BMC Med Educ*, vol. 24, no. 1, p. 41, Jan. 2024, doi: 10.1186/s12909-023-05019-0.

[40]	Q. Wang and T. Du, “Implementation of the college student mental health education course (CSMHEC) in undergraduate medical curriculum: effects and insights,” *BMC Med Educ*, vol. 20, no. 1, p. 505, Dec. 2020, doi: 10.1186/s12909-020-02438-1.

[41]	N. M. Alotaibi *et al.*, “A quasiexperimental study of assessing the impact of stress management program on health science students at Kuwait University,” *Sci Rep*, vol. 15, no. 1, p. 16461, May 2025, doi: 10.1038/s41598-025-01633-8.

[42]	Y. Kotera, A.-M. Edwards, G. Garip, J. Chircop, and M. Aledeh, “Effects of Online Mental Health Classes on Mental Health and Stigma: a Controlled Before-After Study with 1-Month Follow-Up,” *SN Compr. Clin. Med.*, vol. 4, no. 1, p. 172, Jul. 2022, doi: 10.1007/s42399-022-01225-x.

[43]	J. Kelly and M. Birks, “‘ It’s the simple things you do first that start the process of help ’: Undergraduate nursing and midwifery students’ experiences of the Mental Health First Aid course,” *Collegian*, vol. 24, no. 3, pp. 275–280, Jun. 2017, doi: 10.1016/j.colegn.2016.04.004.

[44]	R. Sheriff *et al.*, “Use of interactive teaching techniques to introduce mental health training to medical schools in a resource poor setting,” *Afr. J. Psych*, vol. 16, no. 4, pp. 256–263, Jul. 2013, doi: 10.4314/ajpsy.v16i4.34.
